# Clock-dependent chromatin accessibility rhythms regulate circadian transcription

**DOI:** 10.1371/journal.pgen.1011278

**Published:** 2024-05-28

**Authors:** Ye Yuan, Qianqian Chen, Margarita Brovkina, E Josephine Clowney, Swathi Yadlapalli

**Affiliations:** 1 Cellular and Molecular Biology Program, University of Michigan, Ann Arbor, Michigan, United States of America; 2 Department of Cell and Developmental Biology, University of Michigan, Ann Arbor, Michigan, United States of America; 3 Department of Molecular, Cellular and Developmental Biology, University of Michigan, Ann Arbor, Michigan, United States of America; 4 Michigan Neuroscience Institute, University of Michigan, Ann Arbor, Michigan, United States of America; Universidad de Valparaiso, CHILE

## Abstract

Chromatin organization plays a crucial role in gene regulation by controlling the accessibility of DNA to transcription machinery. While significant progress has been made in understanding the regulatory role of clock proteins in circadian rhythms, how chromatin organization affects circadian rhythms remains poorly understood. Here, we employed ATAC-seq (Assay for Transposase-Accessible Chromatin with Sequencing) on FAC-sorted Drosophila clock neurons to assess genome-wide chromatin accessibility at dawn and dusk over the circadian cycle. We observed significant oscillations in chromatin accessibility at promoter and enhancer regions of hundreds of genes, with enhanced accessibility either at dusk or dawn, which correlated with their peak transcriptional activity. Notably, genes with enhanced accessibility at dusk were enriched with E-box motifs, while those more accessible at dawn were enriched with VRI/PDP1-box motifs, indicating that they are regulated by the core circadian feedback loops, PER/CLK and VRI/PDP1, respectively. Further, we observed a complete loss of chromatin accessibility rhythms in *per*^*01*^ null mutants, with chromatin consistently accessible at both dawn and dusk, underscoring the critical role of Period protein in driving chromatin compaction during the repression phase at dawn. Together, this study demonstrates the significant role of chromatin organization in circadian regulation, revealing how the interplay between clock proteins and chromatin structure orchestrates the precise timing of biological processes throughout the day. This work further implies that variations in chromatin accessibility might play a central role in the generation of diverse circadian gene expression patterns in clock neurons.

## Author summary

The intricate organization of chromatin is crucial for orchestrating precise gene expression patterns throughout various biological processes such as development and differentiation. In this study, we adapted ATAC-seq (Assay for Transposase-Accessible Chromatin) to assay chromatin accessibility genome-wide from FAC (Fluorescence-activated cell)-sorted Drosophila clock neurons at dawn and dusk timepoints over the circadian cycle. Our findings reveal that regulatory elements of hundreds of clock-regulated genes undergo diurnal oscillations in chromatin accessibility, with more accessible chromatin during the activation phase leading to higher transcription, and compacted chromatin during the repression phase leading to gene silencing. Motif analysis further elucidated distinct regulatory motifs associated with chromatin accessibility changes. Specifically, genes exhibiting increased accessibility around dusk were enriched with E-boxes, whereas those with enhanced accessibility around dawn showed enrichment for VP-boxes. Notably, our studies unveiled that these diurnal fluctuations in chromatin accessibility within clock neurons are dependent on the presence of a functional clock, as per^01^ null mutants displayed a loss of these rhythmic patterns. In summary, our studies shed light on the intricate relationship between circadian transcriptional regulation and chromatin accessibility dynamics. These insights underscore the role of chromatin accessibility dynamics in priming the genome for rhythmic gene expression, thereby contributing to the orchestration of circadian rhythms.

## Introduction

Chromatin organization plays a pivotal role in ensuring proper gene expression patterns during development, differentiation, and in response to environmental stimuli [1]. Chromatin can adopt either an "open" or "closed" configuration. Euchromatin is less condensed (open) and is associated with active gene transcription, while heterochromatin is more compact (closed), which makes the DNA less accessible, and is generally associated with gene repression [2]. For example, during the developmental transition from a stem cell to a more specialized cell type, the promoters, enhancers, and other regulatory elements of specific genes necessary for the function of the differentiated cell become more accessible to transcriptional machinery, which enables their expression [3,4]. Conversely, other regions become less accessible, leading to gene silencing. For instance, during mammalian development, key developmental genes like the Hox loci undergo a significant change in chromatin structure, becoming highly accessible during the differentiation process [5].

While the essential role of chromatin accessibility in controlling gene expression during development is widely recognized, much less is known about chromatin accessibility dynamics over the 24-hour circadian cycle and its role in driving circadian rhythms. Circadian clocks are cell-autonomous timekeepers that orchestrate ~24-hour rhythms in the expression of a large number of genes, thereby controlling much of our physiology and behavior, including sleep-wake cycles, and metabolism [6–8]. Prior studies have demonstrated that the core clock transcription machinery recruits a variety of epigenetic remodelers—including histone-modifying enzymes, Polycomb complexes, and ATP-dependent chromatin remodeling enzymes—to orchestrate rhythmic gene expression over the circadian cycle [8]. Further, previous work has identified rhythmic patterns in promoter-enhancer contacts of clock genes over the circadian cycle in mouse liver and kidney cells [9,10]. Additionally, both our past work [11] and that of others [12] have shown that subnuclear location of clock-regulated genes at the nuclear periphery is critical for their rhythmic gene expression.

**Fig 1:**
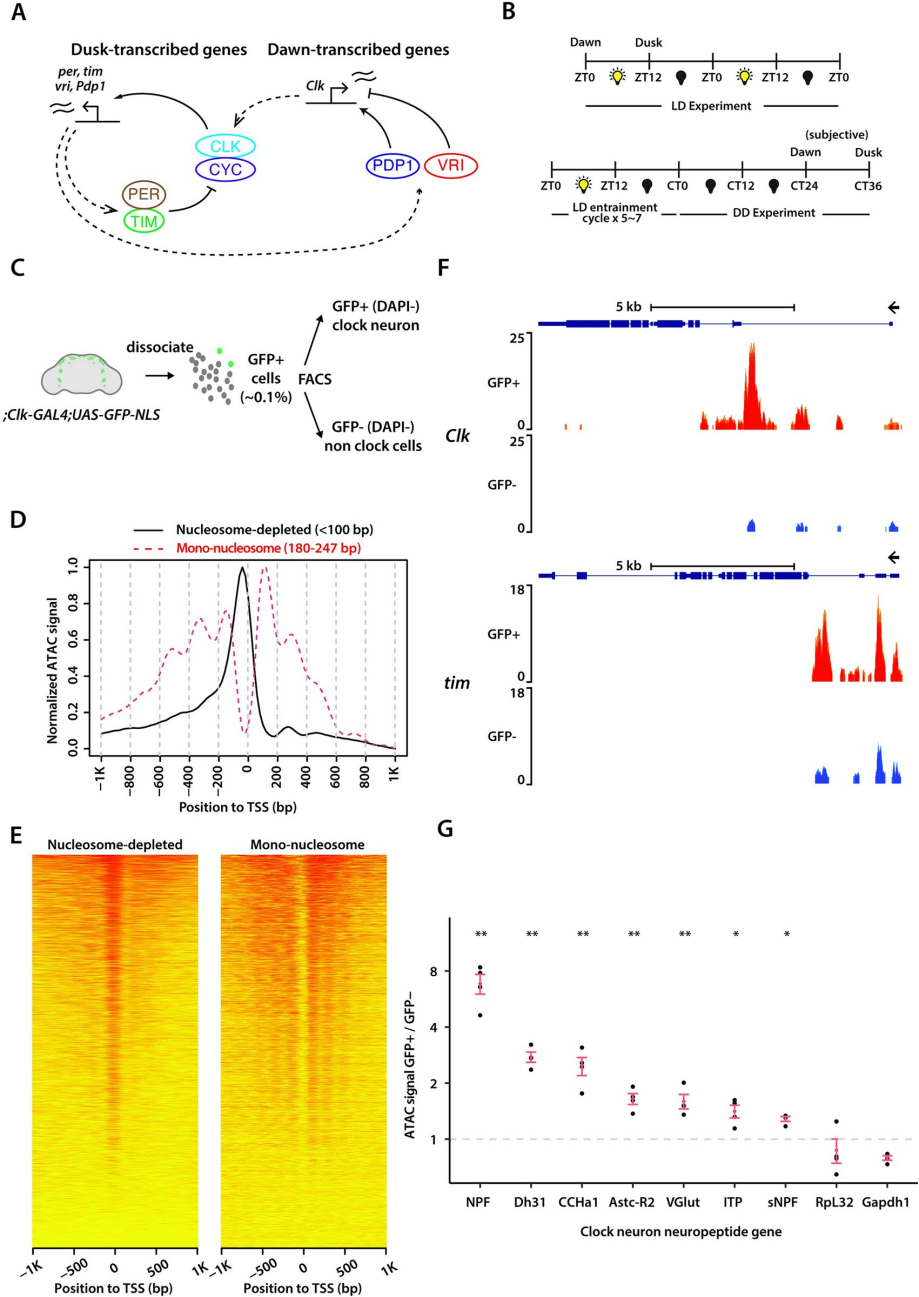
ATAC-sequencing of *Drosophila* clock neurons. (**A**) Schema of the *Drosophila* molecular clock feedback loops. (**B**) Experimental schema. Flies were entrained for 5 days in light-dark cycles (ZT0: lights-on/dawn, ZT12: lights-off/dusk) for LD experiments and then optionally released into complete darkness for DD experiments. (**C**) Overview of clock neuron isolation procedure. Nuclear GFP signal is expressed with *Clk-GAL4* driver and cell suspension was prepared from ~60 brains for each sample. Fluorescence activated cell sorting is used to sort live clock neurons with DAPI as viability marker. GFP-negative cells are sorted as non-clock-neuron control. (**D**) Representative ATAC-signal distribution aligned to transcription start sites (TSS) as reported by ATACseqQC. Fragments are classified into different groups according to alignment length, including the nucleosome-depleted (<100bp) and mono-nucleosome (180~247bp) groups. Clear TSS enrichment of nucleosome-depleted signal and nucleosome ladder are observed. (**E**) Heatmap of normalized ATAC signal distribution aligned to TSS plotted with ATACseqQC. Normalized ATAC signal at different positions relative to TSS (x-axis) were computed against 34920 annotated TSS’s (y-axis). (**F**) Representative ATAC signal pile-up tracks at *Clock* and *timeless* loci. Signal is normalized to total number of reads for each sample and therefore is comparable among samples. Four different replicates are overlaid. Clock neurons (GFP+, DAPI-) show strong ATAC signal in *Clk* and *tim* loci while non-clock cells (GFP-, DAPI-) show significantly lower readout. (**G**) Comparison of ATAC signal (total number of reads over the full-length gene with promoter) at various clock-neuron neuropeptide gene loci between clock-neurons and non-clock cells. While housekeeping genes (*RpL32* and *Gapdh1*) does not show clock-neuron enrichment, many clock-neuron neuropeptide genes are significantly more accessible in clock neurons. For each neuropeptide, a one-way ANOVA test was used to determine whether clock neuron values are different from non-clock cell values. *P < 0.01, **P < 0.001.

Circadian transcription could predominantly be governed by specific protein factors, while the underlying chromatin structure remains stable over the 24-hour circadian cycle. However, an alternative hypothesis is that the clock might actively modulate chromatin accessibility rhythms, thereby producing daily transcriptional oscillations. While earlier studies have investigated chromatin accessibility patterns across the circadian cycle in various model organisms [13–19], the mechanisms by which chromatin accessibility affects circadian rhythms remain less well understood. Here, we utilized *Drosophila melanogaster* as it has a well conserved, yet relatively simple circadian clock system. The *Drosophila* clock network consists of ~150 clock neurons that express clock proteins [20,21] (**S1A Fig**). Circadian clocks in all eukaryotes are based on negative transcription-translation delayed feedback loops [6]. In *Drosophila*, the circadian clock is primarily regulated by two key feedback loops involving the PERIOD/CLOCK and VRILLE/PDP1 proteins [6]. CLOCK (CLK) and CYCLE (CYC) proteins serve as transcriptional activators, binding to the E-boxes (enhancer-box) of clock-regulated genes, including core clock genes such as *period* (*per*), *timeless* (tim), *vrille* (vri), and *pdp1-ε*, and activating their transcription around dusk (**Fig 1A**). After a time-delay, PER and TIM proteins enter the nucleus around dawn, where they function as transcriptional repressors. There, they counteract the activities of CLK/CYC, inhibiting their own transcription as well as the transcription of other clock-regulated genes. The sequential binding of CLK/CYC activators to chromatin around dusk followed by PER/TIM repressors around dawn orchestrates rhythmic gene expression patterns (**Fig 1A**).

The VRI/PDP1 feedback loop forms the second key regulatory circuit. Around dusk, VRI, acting as a repressor, binds to specific sites (VP-box) within the *Clk* enhancer to inhibit its transcription. Conversely, at dawn, PDP1-ε, acting as an activator, binds to these VP-boxes within the *Clk* enhancer to promote its transcription (**Fig 1A**). This alternating binding activity of PDP1-ε (activator) and VRI (repressor) results in the rhythmic expression of *Clk* mRNA [22]. This VRI/PDP1 feedback loop also plays a crucial role in controlling the rhythmic expression of numerous clock output genes, thereby influencing rhythmic behavior [23,24]. These two interlinked feedback loops, PER/CLK and VRI/PDP1, generate gene expression rhythms with opposite phases, where genes regulated by the PER/CLK loop peak around dusk while those regulated by the VRI/PDP1 loop peak around dawn (**Fig 1A**), thereby helping establish a robust circadian rhythm in *Drosophila*.

To investigate the impact of chromatin dynamics on circadian rhythms, here, we adapted ATAC-seq [25] (Assay for Transposase-Accessible Chromatin) to assay chromatin accessibility genome-wide from FAC (Fluorescence-activated cell)-sorted *Drosophila* clock neurons at multiple timepoints over the circadian cycle. Our studies revealed that circadian transcriptional activation and repression are accompanied by dynamic chromatin accessibility states that poise the genome for rhythmic gene expression. Specifically, we found that regulatory elements of clock-regulated genes undergo diurnal oscillations in chromatin accessibility. Motif analysis identified that genes that were more accessible around dusk contained E-boxes, while those that were more accessible around dawn contained VP-boxes. We also found that chromatin accessibility rhythms in clock neurons are completely abolished in arrhythmic *per*^*01*^ null mutants [26]. In summary, these findings highlight the important role of chromatin organization in circadian regulation, demonstrating how the interplay between chromatin accessibility and clock proteins affects circadian rhythms.

## Results

### ATAC-sequencing of *Drosophila* clock neurons

To investigate genome-wide chromatin accessibility within *Drosophila’s* clock neurons, we performed transposase-accessible chromatin sequencing (ATAC-seq) [25], a strategy that enables identification of accessible regions within the chromatin. To isolate and target clock neurons specifically, we used the *Clk-GAL4* driver [27]—which is expressed in almost all Drosophila clock neurons [28]—to express GFP in their nuclei (Fig 1B). The *Clk-GAL4>GFP-NLS* flies displayed normal locomotor-activity rhythms in both light-dark cycles (LD12:12) and constant darkness (DD), displaying ∼24-hour period rhythms and activity peaks around dawn and dusk (**S1B Fig**). To conduct our ATAC-seq experiments, we entrained these flies to a 12:12 LD cycle, and then released them into constant darkness (DD) conditions. Flies were collected either at dawn (ZT0) or dusk (ZT12) in LD, or at subjective dawn (CT24) or subjective dusk (CT36) on the second day of DD (**Fig 1B**). Subsequently, we dissected fly brains, isolated clock neurons using fluorescence-activated cell sorting (FACS), separated nuclei from cytoplasmic content, performed TN5 transposition reaction, and prepared ATAC-seq libraries for paired-end sequencing as described previously [29] (**Fig 1C**). We performed four biological replicates for each condition and timepoint. For each of our experiments, we dissected ~60 brains from both male and female flies which yielded ~2,000 GFP-positive clock neurons. Additionally, we collected ~10,000 GFP-negative cells, representing non-clock cells which comprise a diverse set of neurons and glia in the brain, to serve as our control group. GFP-positive clock neurons constituted 0.1% of cells extracted from fly brains, consistent with previous estimates [28] (**S1C and S1D Fig**).

**Fig 2:**
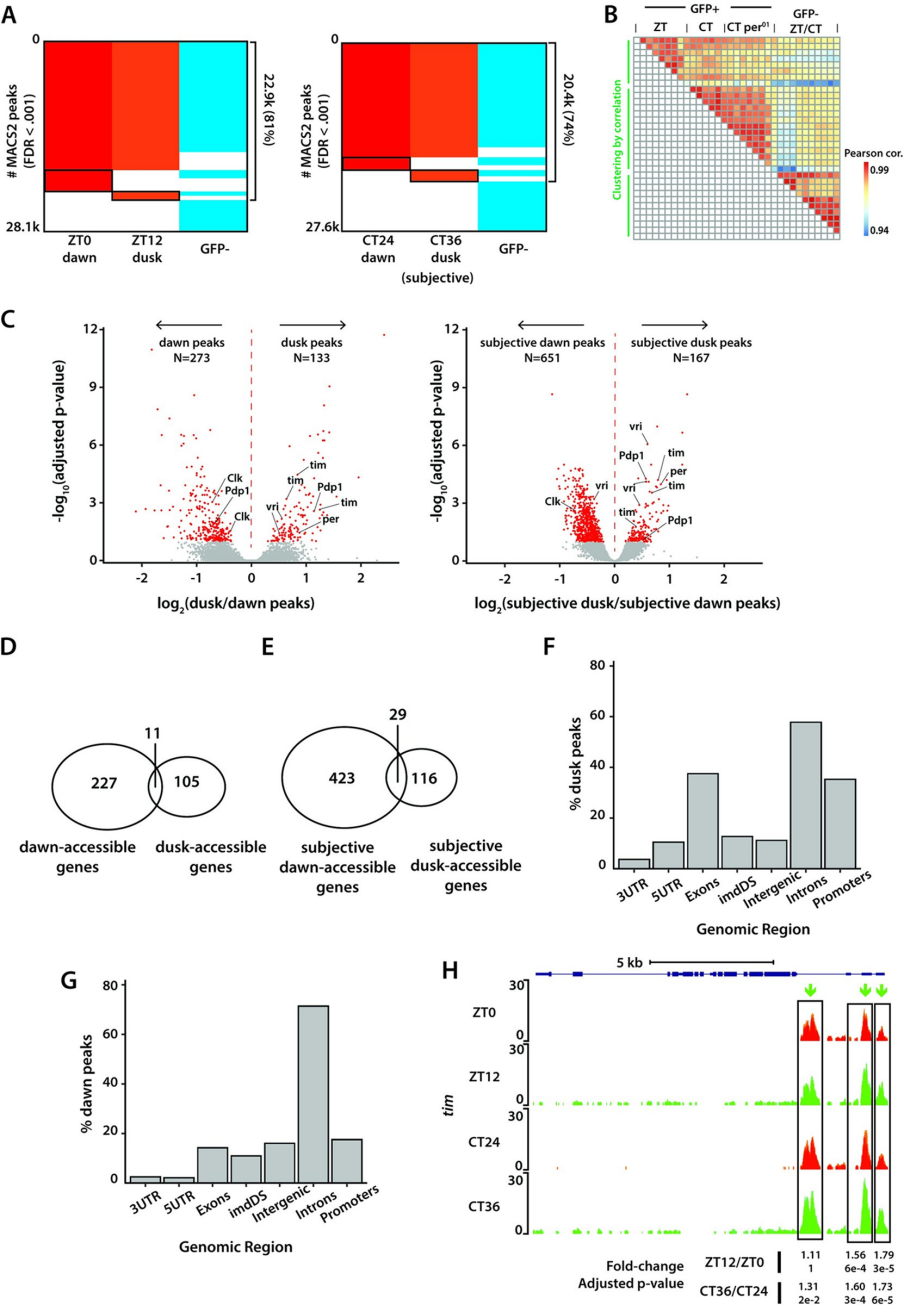
Differential analysis of chromatin accessibility in clock neurons at dawn and dusk. (**A**) Binary heatmaps of MACS2 called peaks for light-dark cycling (LD: ZT0, ZT12) and constant darkness (DD2: CT24, CT36) conditions. MACS2 is used to call distinct ATAC signal peaks for each condition. In total, we identified 28.1k/27.6k peaks under LD and DD conditions. A significant portion of the peaks overlap between GFP-positive clock neurons and GFP-negative cells at both timepoints. Black boxes show peaks called in one time-point only in either LD or DD condition. (**B**) Pairwise correlation heatmap of all samples used in this study. All sample pairs show high correlation (>0.94). Clock neuron samples in ZT0/ZT12 conditions, in CT24/CT36 conditions (including wildtype and *per*^*01*^) and non-clock cell samples in both ZT and CT conditions form three distinct clusters. (**C**) Volcano plots of differential peaks under LD and DD conditions. We identified 406 differential peaks under LD and 818 under DD conditions. Peaks corresponding to core clock genes showed higher accessibility at dusk relative to dawn. vri and Pdp1 have more intricate transcriptional regulation as they possess differential peaks at both dawn and dusk. (**D, E**) Assignment of ATAC peaks to genes by nearest neighbor ranked via chromosome coordinate. The 406 ZT peaks were assigned to 343 distinct genes (~1.2 peaks/gene) and the 818 CT peaks were assigned to 568 genes (~1.4 peaks/gene), showing a mostly bijective mapping. Distribution of dusk (ZT12) peaks (**F**) and dawn peaks (**G**) with respect to known genomic features by the ChIPpeakAnno Bioconductor package. ‘imdDS’ refers to genomic regions immediately downstream (within 1kb) of promoter regions. (**H**) Normalized ATAC signal pile-up tracks of *timeless* locus under LD and DD conditions. *tim* locus has three regulatory elements near its promoter that are more accessible during dusk when ATAC signal along its full-length gene body could also be identified.

After mapping the sequencing reads to the *D*. *mel* genome (*dm6*) and filtering out PCR duplicates that were a by-product of the library amplification process, we were able to achieve a median depth of ~5–10 million unique, high-quality mapped reads per sample. The high Pearson correlation between the biological replicates for each condition (a minimum of 0.94 across all samples) attests to the high reproducibility of our sequencing results and the notable similarity in chromatin profiles across all conditions (**Fig 2B**). Analysis of ATAC-seq signal across gene units from both LD and DD conditions revealed enrichment near the transcriptional start sites (TSSs), denoting the accessible chromatin within promoter regions (**Figs 1D, 1E, and S1E**). We deposited all the ATAC signal pile-up tracks on UCSC Genome Browser (https://tinyurl.com/2ftuhv5p).

To test the validity of our ATAC-seq data, we first assessed whether the chromatin of core clock genes and clock neuron-specific neuropeptide genes exhibited greater accessibility in GFP-positive clock neurons relative to GFP-negative non-clock cells. Our analysis revealed that the promoters of several core clock genes, including *Clk* and *Tim*, exhibited a higher degree of accessibility in GFP-positive clock neurons as compared to GFP-negative cells (**Figs 1F and S2B**). Interestingly, the promoters of other core clock genes, such as *Pdp1* and *Per*, demonstrated accessibility in both GFP-positive clock neurons and GFP-negative cells, suggesting that these promoters are accessible irrespective of their transcriptional status (**S2A and S2C Fig**). In these instances, our data revealed that the regulatory intronic regions of these genes exhibited enhanced accessibility in GFP-positive clock neurons relative to GFP-negative cells (**S2A and S2C Fig**). We have shown pile-up tracks of individual replicates for a few key genes in **S2 Fig**. We note that the pile-up tracks for all genomic loci from all replicates are readily available on the UCSC genome browser at **http://tinyurl.com/2ftuhv5p**.

Moreover, we found that the chromatin of several clock neuron-specific neuropeptides, including NPF (expressed in two LNds), sNPF (expressed in two LNds and four s-LNvs), AstC-R2 (expressed in a single LNd), ITP (expressed in one LNd and the fifth s-LNv), and DH31 and CNMa (expressed in dorsal clock neurons), displayed enhanced accessibility in GFP-positive clock neurons relative to GFP-negative cells (**Figs 1G and S2D**). Notably, many of these neuropeptides are expressed only in a few clock neurons across the whole brain [28], yet our analysis indicates that their gene loci exhibit increased accessibility in clock neurons compared to non-clock cells. These results demonstrate that our ATAC-seq protocol is highly sensitive and can assess genome-wide chromatin accessibility from a relatively small number of FAC-sorted Drosophila clock neurons.

### Chromatin accessibility rhythms in clock neurons

To explore changes in chromatin accessibility within clock neurons, we analyzed ATAC-seq data from experiments conducted at four distinct time points: dusk (ZT12) and dawn (ZT0) under light-dark (LD) conditions, and subjective dusk (CT36) and subjective dawn (CT24) under constant darkness (DD) conditions. Utilizing the MACS2 peak-calling algorithm [30], we identified over 27,000 peaks from GFP-positive clock neurons across all four timepoints, with a false discovery rate (FDR) threshold of < 0.001 (**Fig 2A**). Under both LD and DD conditions, we observed a significant overlap of over 75% in the peaks between GFP-positive clock neurons and GFP-negative cells, as well as between the two timepoints (**Fig 2A**). This overlap likely corresponds to genes integral to basic cellular functions, encompassing housekeeping genes and those related to general neuronal activities. Only a smaller fraction of peaks is unique to each timepoint under both LD and DD conditions. Nevertheless, our results indicate that GFP-positive clock neurons under LD conditions, GFP-positive clock neurons under DD conditions, and GFP-negative non-clock cells under both LD and DD conditions segregate into three distinct clusters, highlighting distinct chromatin accessibility patterns in each group (**Fig 2B**).

To determine if any of these peaks exhibited differential accessibility across dawn and dusk timepoints, we utilized DESeq2 to quantify changes in chromatin accessibility [31], applying an adjusted p-value cut-off of 0.1 (**S3A Fig**). Of the approximately 27,000 peaks observed under LD and DD conditions, we identified 406 differential peaks under LD conditions and 818 differential peaks under DD conditions (**Fig 2C**). Interestingly, there was only a limited overlap in the differential peaks observed under LD and DD conditions, for instance, between dawn and subjective dawn or between dusk and subjective dusk (**S3B, S3C, and S3E Fig**), suggesting unique chromatin landscapes at each of these timepoints. Furthermore, these observations are consistent with previous RNA-seq findings which indicated that, beyond core clock genes, different sets of genes oscillate under light/dark (LD) and constant darkness (DD) conditions [28,32].

Out of the 406 differentially accessible peaks under LD conditions, 273 peaks showed higher accessibility at dawn, while 133 peaks showed higher accessibility at dusk. Out of the 818 differentially accessible peaks identified under DD conditions, 651 peaks showed higher accessibility at subjective dawn, while 167 peaks showed higher accessibility at subjective dusk (**Fig 2C**). To understand peak distribution among annotated genes, we assigned peaks to genes by nearest neighbor on 1D-chromosomal coordinates. The 406 differential peaks found under LD conditions were assigned to 343 distinct genes (~ 1.2 peaks per gene), and the 818 differential peaks found under DD conditions were assigned to 568 genes (~1.4 peaks per gene) (**Fig 2D and 2E**). Interestingly, we observed that the fold-change difference in peak accessibility is overall higher in LD conditions than in DD conditions (**Fig 2C**). We found that dawn-accessible peaks were found mostly in intronic and promoter regions, consistent with previous reports on the chromosomal localization of regulatory elements [33] (**Figs 2F and S3D**), while the dusk-accessible peaks were found mostly in intronic regions (**Figs 2G and S3D**).

Upon examining the chromatin accessibility of core clock genes, we noticed a distinct pattern. Peaks associated with key clock genes like *tim* and *per* exhibited higher accessibility at dusk (ZT12/CT36) (**Figs 2H and S4B**), while peaks associated with the *Clock* gene demonstrated heightened accessibility at dawn (ZT0/CT24) (**S4A Fig**). Specifically, we found that the promoter regions and the intronic regulatory elements of *tim* displayed a higher degree of accessibility at dusk relative to dawn, indicating that these regions transition from an inactive chromatin state to a more active regulatory state over the course of the ~24-hour circadian cycle. Furthermore, we observed a pervasive opening of chromatin across most of the *tim* gene at dusk timepoints, as indicated by the ATAC signal across its gene body (**Fig 2H**). This could be due to high levels of transcription of *tim* gene in all clock neurons. In order to determine whether the widespread chromatin opening is unique to the tim gene or is a common feature, we conducted a differential analysis of ATAC signals across all annotated gene bodies, while excluding peaks identified by MACS2. This approach enabled us to concentrate on pervasive signals rather than those emanating from regulatory elements. Our analysis revealed that ~70 genes exhibit differential pervasive accessibility, with *tim* being the most significant among them (**S1 Table**).

The *per* gene exhibited significant differential peaks primarily in its first intronic region, with increased accessibility at dusk relative to dawn (**S4B Fig**). This intronic region has previously been shown to contain an E-box element which is bound by CLK/CYC proteins [34]. We observed a slight increase in the accessibility of the E-box element within the promoter region of the *per* gene at dusk relative to dawn, however, the observed difference did not reach a statistically significant level (**S4B Fig**). Interestingly, we noted that specific genes, such as vri and Pdp1, exhibited regulatory peaks that were highly accessible at both dusk and dawn. (**S4C, S4D, and S5A Figs**). This pattern may arise from the varied expression of different gene isoforms in specific subsets of clock neurons. For example, from an existing bulk RNA-seq dataset [32], we found that while the short variant of Pdp1 exhibits robust cycling across all clock neuron groups, the long variant is non-rhythmic and is expressed at a consistently high level in a large group of clock neurons (DN1 neurons) throughout the circadian cycle (**S5B Fig**).

**Fig 3:**
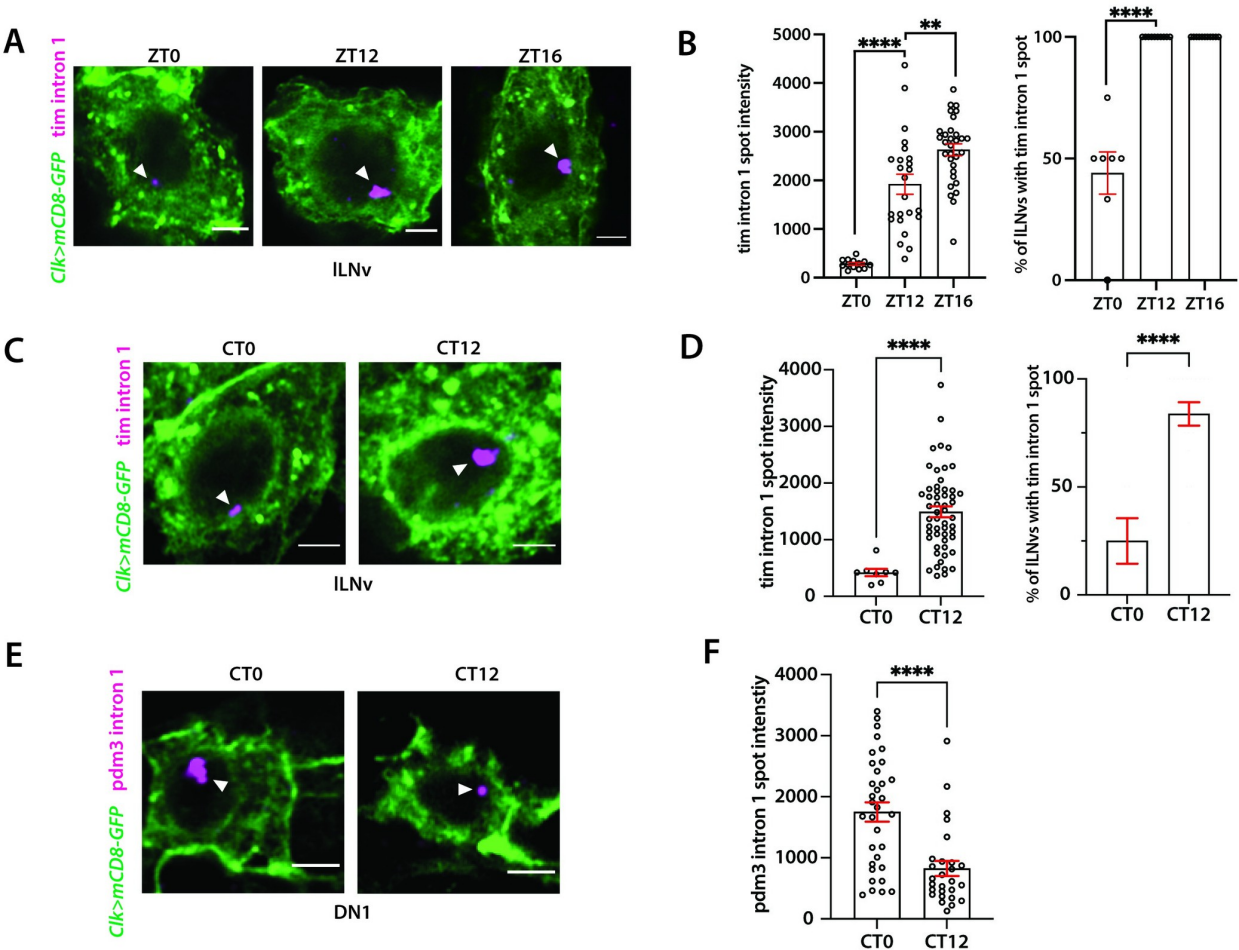
Transcriptional activity of tim and pdm3 genes quantified by intronic HCR-FISH experiments. (**A**) Representative images of *tim* intron HCR-FISH in lLNv clock neurons at dawn (ZT0) and dusk (ZT12, ZT16) timepoints. A specific intron (‘intron 1’) was probed as it yields a single bright spot of the transcription site. (**B**) Spot intensity fluorescence of the *tim* transcription site and percentage of lLNv clock neurons with the transcription spot. Transcription activity of *tim* is higher at dusk compared with dawn, consistent with the ATAC differential analysis. (**C, D**) Representative images and quantification of *tim* intron 1 HCR-FISH at subjective dusk and dawn timepoints. (**E**) Representative images of *pdm3* intron 1 HCR-FISH at subjective dusk and dawn timepoints in DN1 clock neurons. (**F**) Spot intensity fluorescence of the *pdm3* transcription site in DN1 clock neurons. Transcription activity of *pdm3* is higher in DN1’s at subjective dawn compared to dusk, consistent with its chromatin accessibility pattern. As *pdm3* was found to be a cycling gene specific to DN1 neurons by published single cell RNA-seq dataset [28], *pdm3* nascent mRNA was imaged in DN1 neurons instead of lLNvs. The statistical test used was a two-sided Student’s t-test. **P < 0.001. Scale bar: 2μm.

Finally, to test if variations in chromatin accessibility correspond to changes in gene transcription, we employed Hybridization Chain Reaction Fluorescence In Situ Hybridization [35] (HCR-FISH) experiments to visualize nascent *tim* mRNA in lLNv clock neurons at dawn and dusk. We performed HCR-FISH experiments using probes targeting the first intron of *tim*. These experiments revealed a single, highly concentrated spot, indicative of simultaneous nascent RNA synthesis at the transcription site of the *tim* gene (**Fig 3A)**. This was observed as a brighter FISH signal at dusk compared to dawn, suggesting increased transcription of *tim* pre-mRNAs at dusk. Additionally, nearly 100% of lLNv clock neurons displayed a *tim* ‘intron spot’ during the activation phase at dusk, compared to lower than 50% during the repression phase at dawn (**Fig 3A and 3B**). Furthermore, we performed additional HCR-FISH experiments in DD conditions using tim intron 1 probe and observed more intense signal at subjective dusk compared to subjective dawn in lLNvs, consistent with their chromatin accessibility patterns (**Fig 3C and 3D)**. The higher chromatin accessibility of the Timeless gene during its activation phase could enable more sustained interaction with CLK transcription factors, potentially increasing the number and magnitude of transcriptional bursts at dusk. Conversely, during the repression phase, the more condensed chromatin might lead to less consistent and more sporadic CLK binding at the promoter region. As a result, a smaller proportion of lLNv clock neurons—less than half—demonstrate transcriptional bursts, which are also markedly smaller in size. These findings show a strong correlation between chromatin accessibility and transcription activity for *tim* gene.

To further validate our findings, we performed HCR-FISH experiments using probes a targeting an intron of another gene, *pdm3*, which is more accessible at subjective dawn than at subjective dusk. Previous single cell RNA-seq study has shown this gene is rhythmically expressed in DN1 clock neurons [28]. Our HCR-FISH experiments in DN1 clock neurons revealed that the signal from the *pdm3* intron 1 was more intense at subjective dawn compared to subjective dusk in DN1 clock neurons, suggesting higher transcription activity at subjective dawn, which is consistent with our ATAC-seq findings (**Fig 3E and 3F)**. Taken together, our study reveals that the chromatin accessibility of regulatory elements of clock-regulated genes varies between dawn and dusk, with periods of heightened accessibility coinciding with their peak expression times.

### Genes more accessible at dusk contain E-box motifs, while those more accessible at dawn contain VP-box motifs

**Fig 4:**
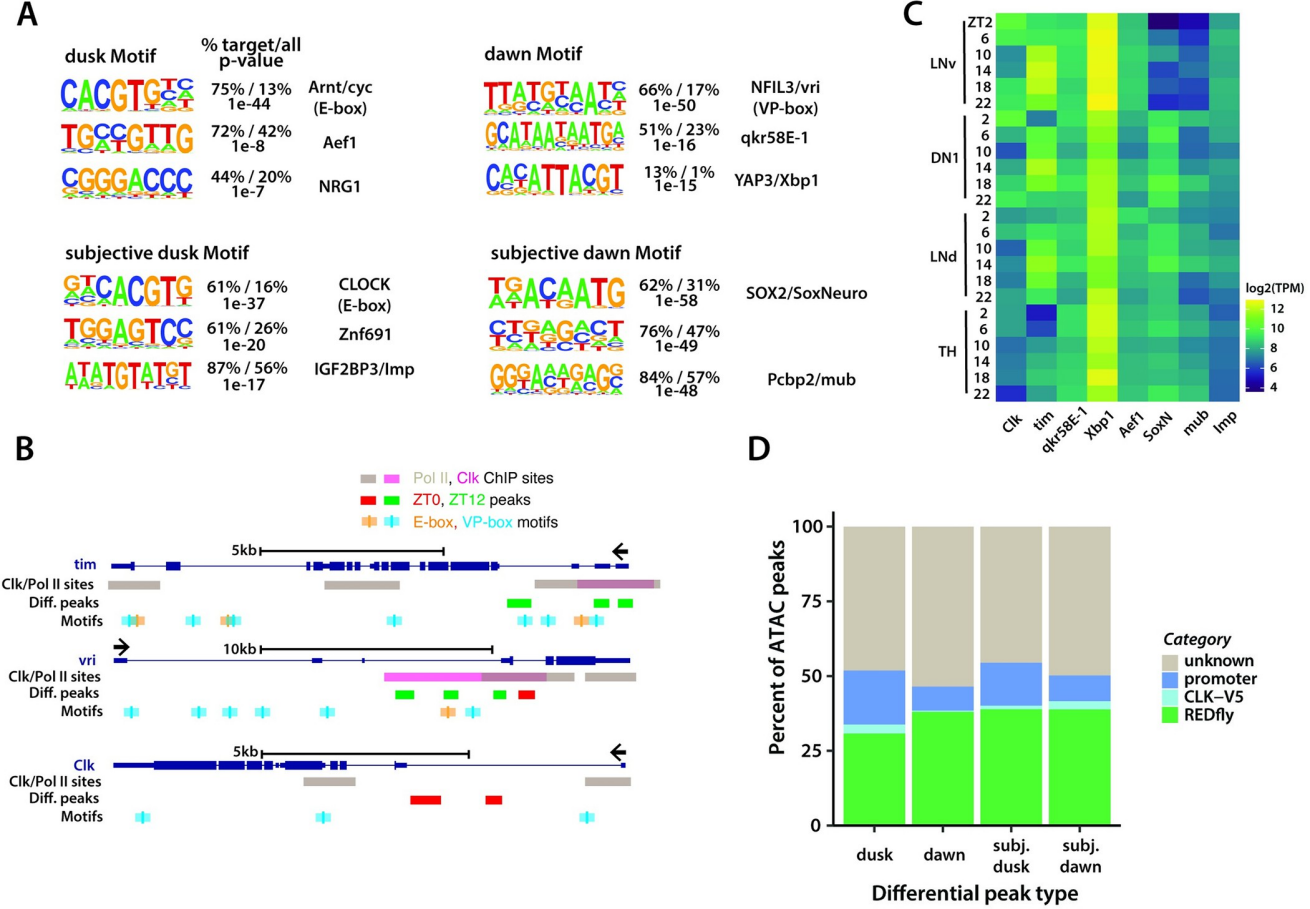
Identification of motifs and analysis of differential peaks. (**A**) De novo motif analysis of differential ATAC peaks. Peaks more accessible at dusk (ZT12/CT36) are highly enriched in E-box sequences, indicating that the corresponding regulatory elements might be directly regulated by the CLK feedback loop. Peaks more accessible at dawn (ZT0/CT24) shows different motif enrichment. ZT0 peaks are enriched in VP-box sequences while CT24 peaks are instead enriched in HMG-domain transcription factor SoxNeuro motif. (**B**) mRNA-seq expression profile of identified motif proteins in different clock neuron groups (LNv, DN1, LNd) and a control non-clock cell group (TH). Analysis was performed with publicly available data (32). (**C**) Illustration of E-box and VP-box motif sites along core clock genes *tim*, *vri*, and *Clk*. RNA polymerase II (Pol II) and CLK binding sites [42] and ATAC differential peaks (from this study) are shown. (**D**) Overlap analysis of differential ATAC peaks against known regulatory elements. ATAC peaks are first overlapped with a public database of Drosophila cis-regulatory elements [43] (REDfly). For the peaks that do not overlap, they are then overlapped with CLK-binding regions identified by a previous microarray study [42]. Finally, the unannotated peaks are overlapped with promoter regions. Overall, ~50% of differential ATAC peaks correspond to known regulatory elements.

To identify potential transcription factors that might bind to the differentially accessible peaks within clock neurons, we conducted both de novo and known motif discovery analyses using the HOMER tool [36]. Intriguingly, we observed a substantial overrepresentation of the E-box motif [37, 38], with more than 70% prevalence, in peaks that were more accessible at dusk (**Fig 4A**). Conversely, the VP-box motif [22] was predominantly identified in peaks that were more accessible at dawn (**Fig 4A**). The E-box motif is the target of the CLOCK and Arnt-like transcription factors, homologs to the *Drosophila* CLOCK and CYCLE proteins [37,38], while the VP-box motif can be bound by the transcription factors NFIL3 and DBP, homologs to the *Drosophila* VRILLE and PDP1 proteins [22]. Our findings indicate that peaks exhibiting increased accessibility at dusk predominantly correlate with genes regulated by the PER/CLK feedback loop, such as *per*, *tim*, *vri*, and *Pdp1*. Conversely, peaks with enhanced accessibility at dawn tend to be associated with genes regulated by the VRI/PDP1 feedback loop, such as *Clk*. These chromatin accessibility patterns correspond well with known phases of circadian gene expression [28].

Beyond the E-box and VP-box motifs, we discovered additional motifs linked specifically to peaks that were more accessible either at dusk or dawn. By analyzing a previously published RNA-seq dataset [28], we found that a majority of these transcription factors are expressed across various clock neuron subsets, implying that they could play a role in modulating the diurnal rhythms of essential biological functions (**Fig 4B**). Specifically, at dawn, we found motifs associated with qkr58E-1, a central component of the spliceosome machinery, and Xbp1, a central component in the unfolded protein response (**Fig 4A**). Our findings are particularly striking as past research has demonstrated that alternative splicing of numerous mRNAs crucial for processes like metabolism, cell cycle, apoptosis, and cell proliferation as well as unfolded protein response are regulated rhythmically over the circadian cycle [39,40]. Collectively, our data hint at a coordinated upregulation of vital pathways like alternative splicing and the unfolded protein response during dawn.

Under constant darkness (DD) conditions, we observed a significant enrichment of the E-box motif in peaks that were more accessible at subjective dusk, similar to our previous observations at dusk in LD conditions (**Fig 4A**). However, while in LD conditions we noted a VP-box motif enrichment at dawn, under DD conditions, SOX2 was the predominant motif at subjective dawn (**Fig 4A**). Additionally, we performed known motif analyses, but we couldn’t identify a notable presence of the VP-box motif in peaks with heightened accessibility at this subjective dawn period, suggesting that the VRI/PDP1 feedback loop might be less active or differently regulated under constant darkness conditions. Notably, recent studies have shown that SOX2 is expressed in adult SCN neurons and is essential for the expression of neuropeptides and maintaining ~24-hour locomotor rhythms [41]. Our results underscore the potential pivotal role of SOX2 in modulating circadian rhythms, especially under constant darkness conditions.

Next, we compared our ATAC-seq profiles with previously published CLK and RNA polymerase II (Pol II) ChIP-seq data from *Drosophila* clock neurons [42]. We observed that differential peak regions for core clock genes either align directly with or are closely adjacent to CLK and Pol II binding sites (**Fig 4C**). However, generally, less than 10% of peaks overlapped with the CLK/Pol-II regions identified by [42]. Notably, the differential ATAC peaks are predominantly located far from the CLK/Pol-II sites, with a median distance of ~30kb (**S6 Fig, see** **Discussion**). Furthermore, we investigated the overlap of differential peaks identified in our ATAC-seq experiments with other established regulatory elements. This included cis-regulatory elements highlighted in a prior circadian study [42] as well as entries from the REDfly database, which is a dedicated repository for transcriptional regulatory elements in Drosophila [43]. We also performed overlap analysis between our differential ATAC-peaks overlap with characterized insulator sites bound by CTCF, SU(HW), BEAF-32 [44–46]. We found that 11.3% of the subjective dusk/dawn peaks and 4.2% of the dusk/dawn peaks overlap with insulator sites. Overall, our analysis revealed that ~50% of the differential peaks overlap with known regulatory elements (**Fig 4D and S2 Table**). Our results reveal that genes with heightened accessibility at dusk are predominantly regulated by the PER/CLK loop through E-box motifs [37,38], whereas genes with increased accessibility at dawn likely fall under the regulatory domain of the VRI/PDP1 loop, mediated by VP-box motifs [22].

### Chromatin accessibility rhythms correlate with mRNA expression rhythms observed in clock neurons

**Fig 5:**
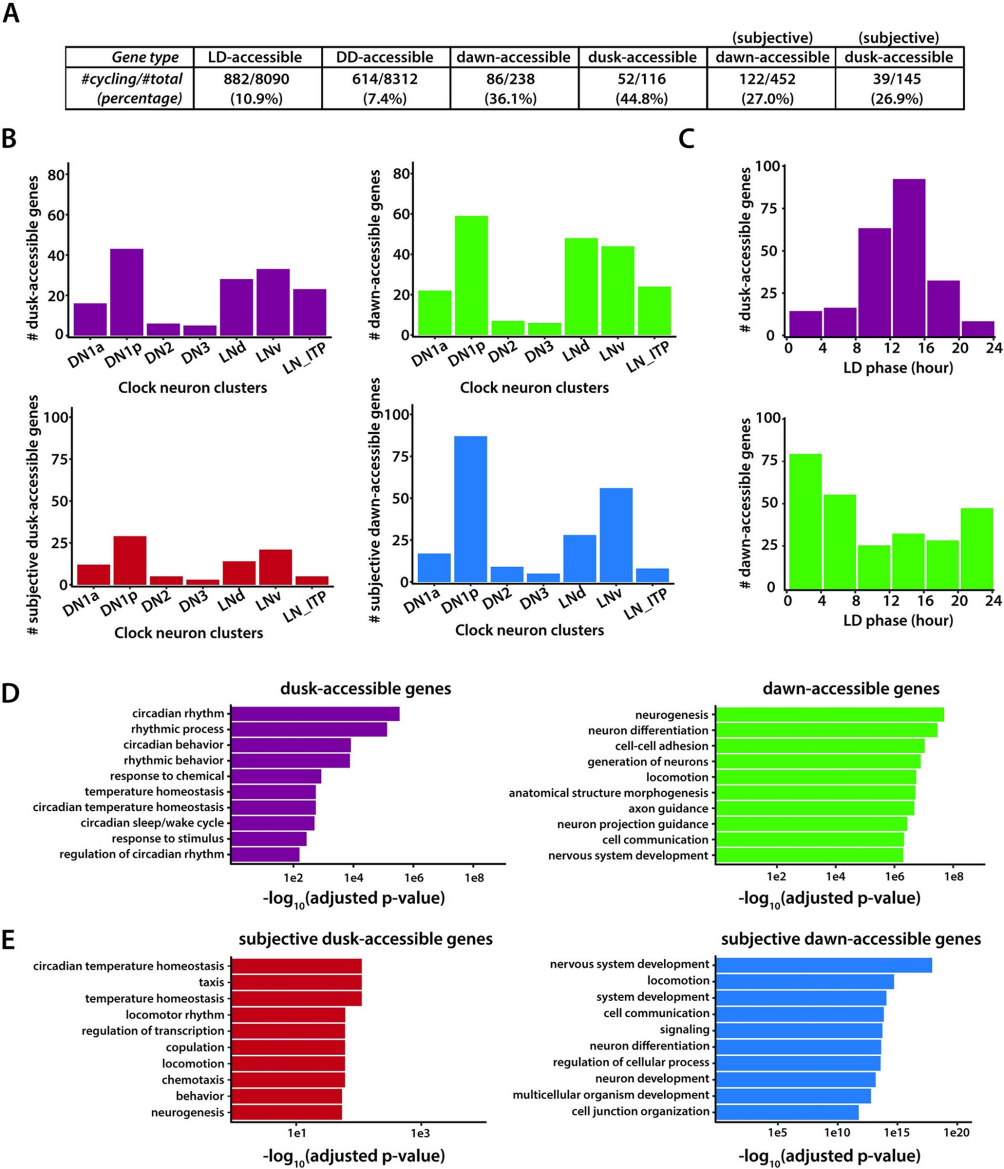
Correlation between gene expression and chromatin accessibility in clock neurons. (**A**) Table showing overlap between scRNA-seq data set [28] and our ATAC-seq dataset. For genes with any ATAC peak (i.e., genes that are accessible in clock neurons), a small percentage of them are found to be cycling in scRNA-seq dataset (10.9% and 7.4% under LD and DD conditions, respectively). For genes with differential ATAC peaks, overlap percentage increases to 36%/45% under LD and 27% under DD conditions. Analysis was performed with publicly available data [28]. (**B**) Distribution of genes with differential ATAC peaks under LD and DD conditions among all the scRNA-seq clock neuron clusters. (**C**) Distribution of phases for peak expression for genes that were more accessible at dawn or dusk. Dawn-accessible genes tend to reach peak transcript levels around ZT0, while dusk-accessible genes tend to reach peak transcript levels around ZT12, indicating strong correlation between chromatin accessibility and transcription. Individual genes may be cycling in multiple clock neuron clusters with different phases and all phases are plotted. (**D**) Ontology enrichment of genes that were more accessible at dusk or dawn. (**E**) Ontology enrichment of genes that were more accessible at subjective dusk or subjective dawn. To remove trivial enrichment terms from pan-neuronal genes, a custom background set of all clock-neuron accessible genes is used.

To assess the relationship between rhythms in chromatin accessibility and gene expression within clock neurons, we compared our ATAC-seq findings with data from a previously published single-cell RNA-seq study [28]. Our analysis revealed that between 30–40% of genes displaying rhythms in chromatin accessibility also show cycling patterns in their transcript levels across different clock neuron clusters (**Fig 5A**). Remarkably, despite our ATAC-seq being a bulk procedure that captures all clock neurons, we were able to identify rhythmic chromatin accessibility in genes expressed specifically within smaller subsets of clock neurons. Furthermore, our data highlighted a significant presence of genes linked to the DN1p clock neuron group (**Fig 5B**). This aligns with prior observations indicating a higher prevalence of DN1p neurons in the brain compared to other clock neuron groups [28].

Importantly, our ATAC-seq findings reveal a congruence between the phases of higher chromatin accessibility and peak RNA expression as reported in prior RNA-seq studies [32]. Specifically, while genes with heightened chromatin accessibility at dusk (ZT12) typically achieve their peak mRNA expression between ZT12 and ZT16, those most accessible at dawn (ZT0) tend to peak between ZT0 and ZT4 (**Fig 5C**). This coherence between enhanced chromatin accessibility and peak gene expression underscores the significance of chromatin dynamics in orchestrating rhythmic gene transcription in clock neurons. The variations in the phases of peak mRNA expression for some genes that we observed could be due to the influence of post-transcriptional regulation [47,48].

Finally, gene ontology (GO) enrichment analysis revealed that genes with heightened chromatin accessibility at dusk show a significant enrichment for circadian rhythms (**Fig 5D and 5E**). In contrast, genes specifically more accessible at dawn were predominantly associated with processes like neuronal structure morphogenesis and axon guidance (**Fig 5D and 5E**), hinting at an upregulation of these processes in the day’s early hours. These findings are consistent with previous research demonstrating that small ventrolateral neurons (sLNvs), a group of clock neurons, experience daily structural changes leading to more intricate branched projections during the early day compared to the early night [49]. To sum up, we found that phases of heightened chromatin accessibility in clock neurons align closely with phases of peak gene expression, underscoring the intricate relationship between chromatin dynamics and rhythmic gene expression within clock neurons.

### Chromatin accessibility rhythms are abolished in the arrhythmic *per*^*01*^ null mutants

**Fig 6:**
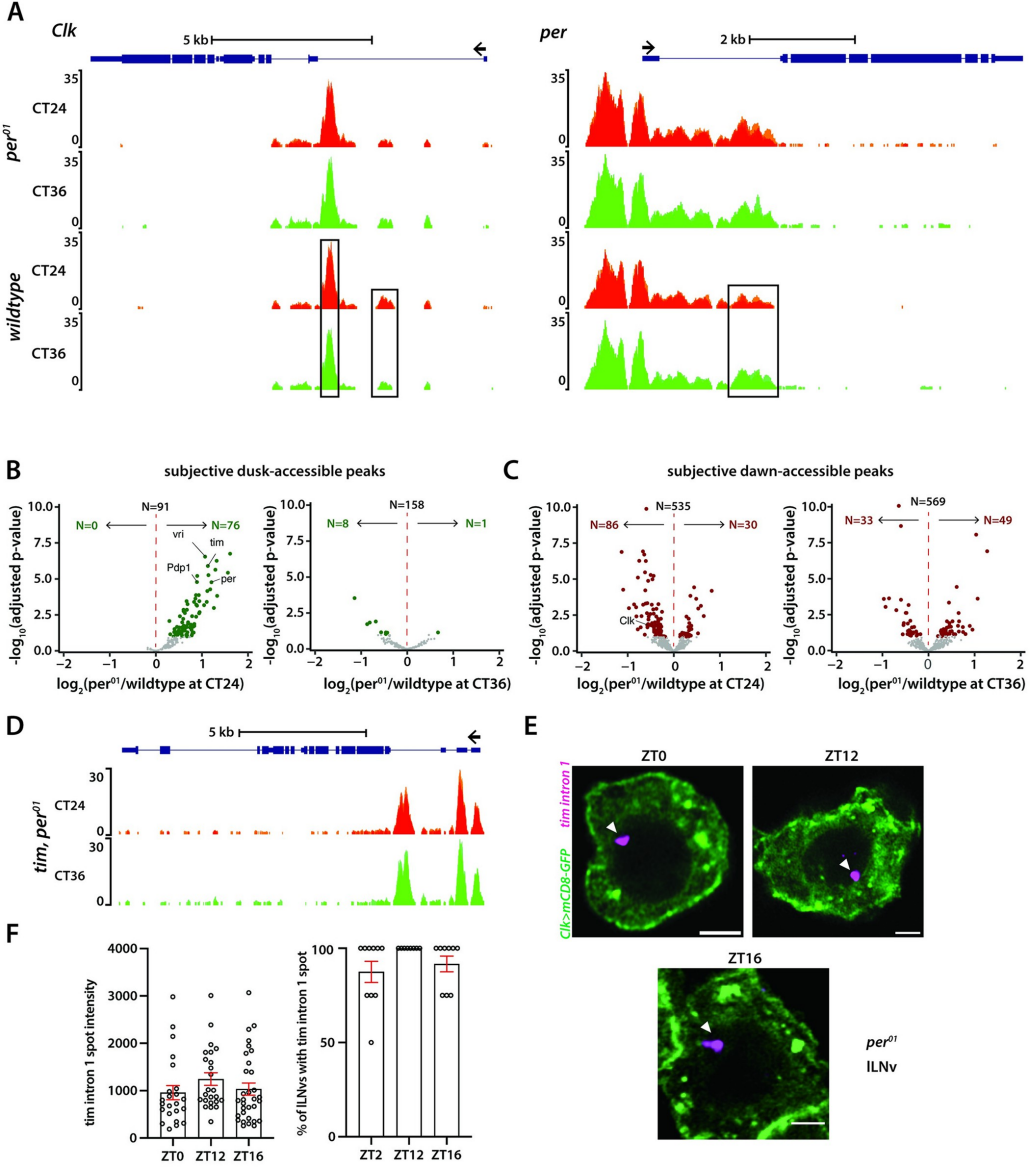
Chromatin accessibility rhythms are abolished in arrhythmic *per*^*01*^ null mutants. (**A**) ATAC signal pile-up tracks of *Clk* and *per* gene loci in clock neurons from *per*^*01*^ mutants at subjective dawn and dusk. As showcased here and by a genome-wide differential analysis, no differential accessibility is identified between subjective dawn and subjective dusk in *per*^*01*^ mutants. Peaks that are differentially accessible in wildtype are marked by black boxes. (**B-C**) Volcano plots of differential peaks comparing *per*^*01*^ to wildtype at subjective dawn and subjective dusk timepoints. (**D**) ATAC signal pile-up tracks of *tim* locus in clock neurons from *per*^*01*^ mutants at subjective dawn and dusk. (**E-F**) Representative images of *tim* intron HCR-FISH (E) and quantification of fluorescence intensity and percentage of lLNv clock neurons with the transcription spot in *per*^*01*^ mutants at dawn (ZT0) and dusk timepoints (ZT12, ZT16) (F). The statistical test used was a two-sided Student’s t-test, no significance was detected. Scale bar: 2μm.

We next sought to determine whether a functional clock is required for generating rhythms in chromatin accessibility in clock neurons. To address this, we performed ATAC-seq experiments on FAC-sorted clock neurons from *per*^*01*^*;Clk-GAL4;UAS-GFP-NLS* flies [26] at two timepoints on the second day of constant darkness (DD2), subjective dawn (CT24) and subjective dusk (CT36). Notably, in stark contrast to the wild-type condition where we observed 818 differentially accessible chromatin regions between subjective dawn and subjective dusk, we detected no differentially accessible regions between these timepoints in the *per*^*01*^ mutant flies. Furthermore, the ATAC profiles for core clock genes in the *per*^*01*^ mutants at both timepoints closely resembled the wildtype’s subjective dusk profile, indicating that these genes remain more accessible in the absence of a functional clock (**Fig 6A and 6D**).

Of the 818 peaks with differential accessibility found in wild-type settings, 167 peaks were more open at subjective dusk, whereas 651 were more open at subjective dawn (**Fig 2C**). In *per*^*01*^ mutants, many of these 167 subjective dusk peaks, especially those linked to core clock genes, exhibited increased accessibility at CT24 compared to the wildtype (**Fig 6B**). Notably, almost none showed reduced accessibility in the *per*^*01*^ mutants compared to wildtype at this timepoint. Moreover, when comparing the *per*^*01*^ mutants to wild-type conditions at subjective dusk (CT36), there were no significant differences in chromatin accessibility (**Fig 6B**). Together, our data suggest that the PER protein plays a pivotal role in facilitating dawn chromatin compaction, and clock-regulated genes remain in a consistently "open" state throughout the circadian cycle in the absence of a functional clock.

Next, we examined how the 651 peaks which were more accessible at subjective dawn in wild-type flies are affected in *per*^*01*^ mutants. We observed that peaks associated with the *Clk* gene displayed diminished accessibility at CT24 in the absence of PER, consistent with previous findings of reduced *Clk* mRNA levels in *per*^*01*^ mutants [50] (**Fig 6C**). Yet, a comparative analysis between wildtype and *per*^*01*^ conditions for other peaks did not follow a consistent pattern; some peaks displayed heightened accessibility in *per*^*01*^, while others were less accessible (**Fig 6C**). Thus, while PER protein plays a specific and significant role in regulating chromatin accessibility of *Clk*, its regulatory control over other dawn-activated genes seems more nuanced, suggesting a possible indirect and context-dependent regulatory mechanism.

As our findings indicate that the lack of PER renders the chromatin of clock-regulated genes more accessible, we asked if this leads to higher transcription of clock-regulated genes at both dawn and dusk. To test this, we employed HCR-FISH to visualize nascent *tim* mRNA transcripts in *per*^*01*^ background. Unlike the wild-type scenario (**Fig 3B and 3D**), we observed no significant difference in the fluorescence intensity of the *tim* ‘intron spot’ between dusk and dawn timepoints in *per*^*01*^ mutant flies (**Fig 6E and 6F**). Moreover, nearly all clock neurons in *per*^*01*^ mutant flies exhibited a *tim* ‘intron spot’ at both dawn and dusk timepoints (**Fig 6E and 6F**), suggesting that *tim* is expressed at high levels throughout the day in the absence of a functional clock. To sum up, our results indicate that a functional clock is essential for the chromatin compaction of clock-regulated genes during the repression phase, which is required for generating ~24-hour gene expression rhythms.

### Differential chromatin accessibility in non-clock cells

**Fig 7:**
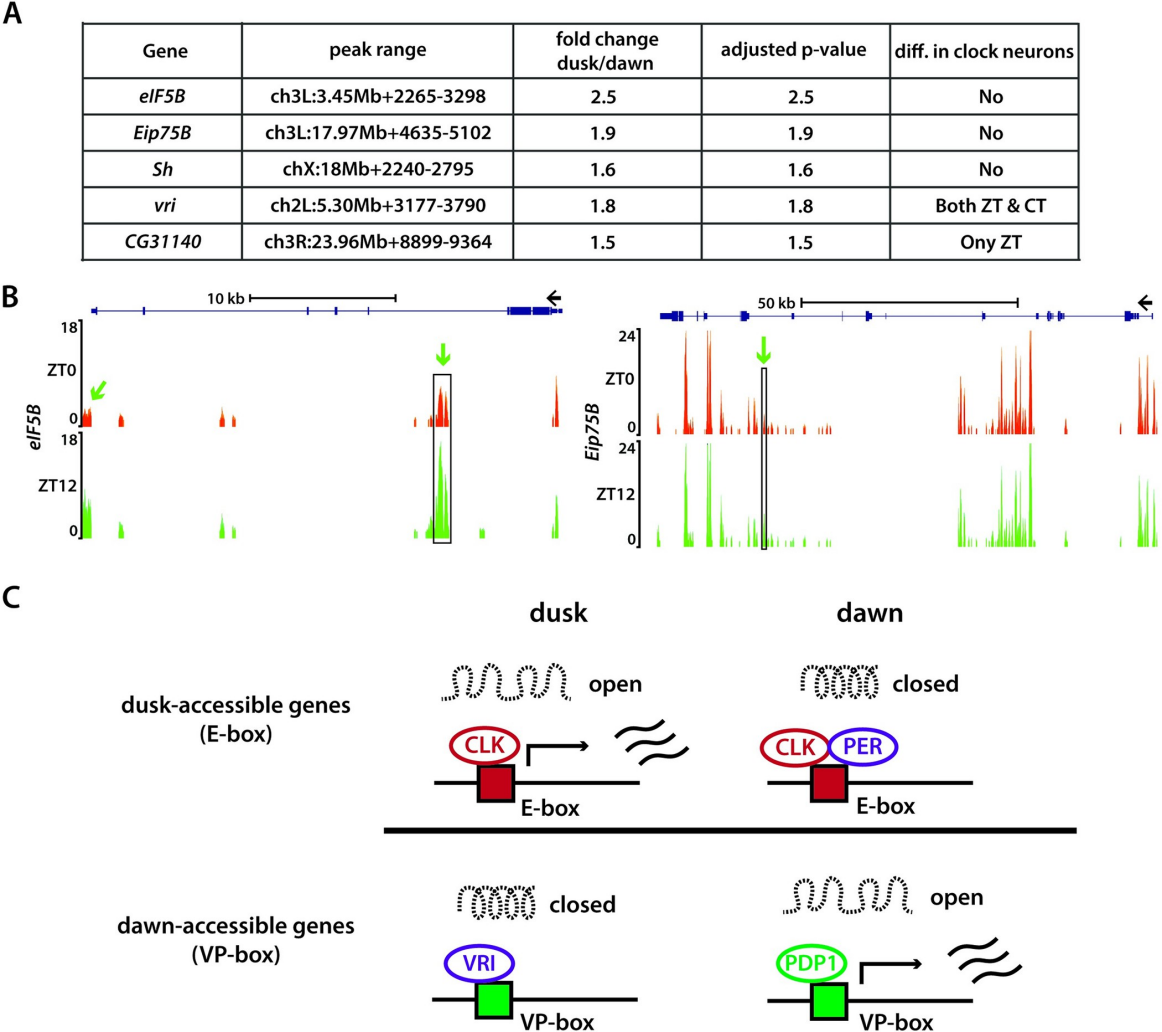
Differential chromatin accessibility in non-clock cells and model. (**A**) Summary table of differential peaks identified in non-clock cells at dusk compared to dawn. (**B**) ATAC signal pile-up tracks of *eIF5B* and *Eip75B* gene loci in non-clock cells at dawn and dusk. Differentially accessible peaks are marked in black boxes. (**C**) Summary of our model. Chromatin accessibility of regulatory elements of clock-regulated genes varies at dawn and suck timepoints over the circadian cycle. E-box containing genes, such as *per* and *tim*, were typically more accessible at dusk, while VP-box containing genes, such as *Clk*, were typically more accessible at dawn.

We asked whether we could identify any differentially accessible peaks between dawn and dusk timepoints in GFP-negative cells, which include a diverse set of neurons and glial cells in the brain. Interestingly, we detected a small group of differentially accessible peaks that were more accessible at dusk (ZT12), a period typically associated with sleep in flies, in comparison to dawn (ZT0) (**Fig 7A and 7B**). This suggests that these regulatory elements might be more accessible across a broad range of neurons in the brain during dusk. These peaks are associated with a select group of genes, including the eukaryotic translation initiation factor (*eIF5B*), Ecdysone-induced protein 75B (*Eip75B*), Shaker (*Sh*), Vrille (*vri*), and *CG31140* (**Fig 7A and 7B**). Given the crucial role of eIF5B in translation initiation, our results suggest a potential upregulation in translation activity during dusk, which aligns with previous studies that showed an accumulation of messenger RNAs and proteins related to translation during the sleep phase [51]. The vrille gene, required for both development [52] and circadian rhythms [53], encodes a transcriptional repressor and is known to be widely expressed in the brain. We found that ATAC-peaks linked to the vrille gene exhibited heightened accessibility at dusk not just within clock neurons but also in other brain neurons (**S4D Fig**). We also observed increased accessibility at dusk in sites connected to CG31140, a lipid kinase essential for the ATP-driven phosphorylation of diacylglycerol (DAG) to yield phosphatidic acid [54], hinting at temporal regulation of lipid signaling pathways.

Interestingly, our analysis revealed enhanced accessibility in the regulatory regions of two key sleep-regulating genes, Shaker and Eip75B [55–57], during dusk—a period when flies are naturally asleep—compared to dawn. The Shaker gene, responsible for encoding a voltage-gated potassium channel [58], plays a critical role in modulating the excitability of neurons and mutations in Shaker have been linked to disrupted sleep behaviors, including shorter sleep and more fragmented sleep patterns [55,56]. Recent studies have shown that the ecdysone receptor (EcR) and its downstream nuclear hormone receptor Eip75B (E75) influence the timing and duration of sleep [57]. Furthermore, Eip75 has been identified as a high-confidence cycling gene in several RNA-seq studies [59,60], with its mRNA expression phase reported to vary across different clock neuron types, spanning from ZT6 to ZT12. Intriguingly, our research reveals that the regulatory regions of Eip75 also display diurnal accessibility patterns in non-clock cells, being twice as accessible at dusk as compared to dawn.

To further delve the question of differential chromatin accessibility in GFP-negative cells, we focused on testing whether the transcriptional activity of the Shaker gene exhibits variation between dawn and dusk in non-clock cells. Specifically, we employed the fru-GAL4>mCD8-GFP marker to label courtship neurons in the brain [29]. We note that a small portion of fru-positive neurons are also recognized as clock neurons [28]. Nevertheless, we made certain that our imaging was confined to the non-clock neurons identified by the fru-GAL4 driver, leveraging their distinct anatomical features for precise selection. We designed HCR-FISH probes targeting each of the seven different introns within the Shaker gene, and identified that probes targeting Shaker intron 4 revealed a unique transcription spot within the nucleus. Our findings indicate that the intensity of the FISH signal for the Shaker intron does not significantly vary between dawn and dusk in fru-positive neurons (**S7 Fig**). This suggests that the pre-mRNA levels of Shaker do not oscillate from dawn to dusk in this specific set of non-clock neurons (Fru-positive neurons). It is possible that other subset of neurons might display rhythms in Shaker transcription from dawn to dusk, which remains to be determined. Future studies could shed light on whether the regulatory regions of the Shaker gene exhibit differential accessibility at dawn versus dusk within a particular subset of brain cells, and if this variation contributes to rhythmic transcription patterns.

## Discussion

Biological clocks are regulated by clock proteins that participate in complex feedback loops, which ensure the timely expression and repression of specific genes that aligns with the day-night cycle. Our study introduces an additional layer of complexity to this understanding. We have unveiled how clock proteins coordinate rhythms in chromatin accessibility across the regulatory elements of numerous clock-regulated genes in *Drosophila* clock neurons, which might be vital for the establishment of approximately 24-hour rhythms in mRNA expression (**Fig 7C**). Our results suggest that differences in chromatin accessibility could be a key factor that contributes to the observed heterogeneity in circadian gene expression within clock neurons. A recent study on mouse liver cells has demonstrated similar dynamic changes in the accessibility of the liver genome throughout the day [61]. This convergence of findings—both in Drosophila and mammals—underlines the universality and importance of chromatin accessibility in shaping circadian rhythms.

### Chromatin accessibility rhythms in clock neurons and non-clock cells

This study represents the first investigation into the dynamics of chromatin accessibility genome-wide in FAC-sorted Drosophila clock neurons. Typically, ATAC-seq requires tens of thousands of cells to assess chromatin accessibility. Considering the Drosophila brain has only ~150 clock neurons, conducting ATAC-seq experiments with such a scarce cell population posed significant technical challenges. In this study, we successfully overcame these challenges and conducted ATAC-seq experiments on ~2000 FAC-sorted Drosophila clock neurons. Our study identified two distinct gene sets based on their chromatin accessibility patterns: one set has regulatory elements that were more accessible at dusk, while the other set has regulatory elements more accessible at dawn. Motif analysis revealed that genes with heightened accessibility at dusk possessed E-box motifs [37,38], suggesting they might be governed by the PER/CLK loop. In contrast, genes more accessible at dawn possessed VP-box motifs [22], suggesting they might be controlled by the VRI/PDP1 loop. In addition to the E-box and VP-box motifs, we identified other motifs uniquely associated with chromatin peaks exhibiting heightened accessibility at either dusk or dawn. Intriguingly, peaks with heightened accessibility at dawn were associated with motifs tied to alternative mRNA splicing and the unfolded protein response. These findings align with prior studies and emphasize the cyclical regulation of processes like alternative splicing and the unfolded protein response over the circadian cycle [39,40].

In our analysis of chromatin accessibility in GFP-negative (non-clock) cells, we made an intriguing observation that regulatory regions of a small set of genes exhibited increased accessibility at dusk compared to dawn. This suggests a unique temporal regulation in the chromatin landscape of these specific genes across non-clock cells in the brain during the day-night cycle. It’s worth noting that the opposite scenario–peaks being more accessible at dawn than at dusk in non-clock cells–was not observed. Given that flies are diurnal creatures, sleeping during the lights-off phase from ZT12 to ZT0, it would be intriguing to investigate whether these changes in chromatin accessibility contribute to the regulation of sleep patterns, behaviors, or other physiological processes during their sleep phase. Furthermore, it’s intriguing to consider how these rhythms might emerge without a traditional clock mechanism and to discern the influence of ambient light, or its absence, on these chromatin accessibility patterns.

### Chromatin accessibility correlates with peak mRNA expression in clock neurons

The relationship between chromatin structure and gene expression can be intricate and multifaceted, as various factors, such as the timed presence of specific transcription factors during the circadian cycle, epigenetic alterations, and the spatial organization of the nucleus, might all modulate gene expression. Interestingly, our research demonstrates that a significant 30–40% of genes displaying circadian rhythms in chromatin accessibility also show oscillations in transcript levels among various clock neuron clusters. This underscores the crucial interplay between chromatin dynamics and gene transcription in the case of clock-regulated genes in clock neurons. This is especially striking considering the central role post-transcriptional regulation plays in shaping ~24-hour circadian rhythms [47,48].

Circadian regulation has been shown to be largely cell- and tissue-specific in both *Drosophila* and mammalian clock neurons [28,62]. In most cases, only transcripts of core clock genes and a handful of other genes demonstrate consistent cycling across all clock neuron groups. While many mRNAs showed cyclical patterns in single-cell RNA-sequencing studies [28], these patterns were often absent in the wider context of fly head or brain mRNA [63]. However, our ATAC-seq method proved effective in detecting rhythmic chromatin accessibility in gene regulatory regions, even if rhythmic expression was limited to a small subset of clock neurons.

We performed analysis to assess how our differential ATAC-seq peaks overlapped with regulatory elements characterized in other studies including CLK/Pol-II regions [42], REDfly database [43], and insulator sites [44–46]. Our analysis revealed that ~50% of the differential peaks overlap with known regulatory elements, however, only ~10% of peaks overlapped with the CLK/Pol-II regions. The minimal overlap between these two sets of peaks is intriguing. The discrepancy could arise from the fact that our data were sourced from FACS-sorted clock neurons, whereas the datasets involving CLK/Pol-II were derived from whole head extracts that included cells expressing ectopically tagged CLK protein and employed microarray technology [42].

### Transcriptional dynamics of Timeless gene

Prior research has demonstrated that the frequency of transcriptional bursting is dependent on the rate of successful binding events at sites near the transcription start site [64]. Prolonged binding of transcription factors at a specific promoter can increase the probability of forming a pre-initiation complex, potentially triggering several rounds of transcription and thus enhancing both the frequency and magnitude of transcriptional bursts. Increased chromatin accessibility of the Timeless gene during its activation phase might facilitate extended binding of CLK transcription factors, thereby elevating both the frequency and size of transcriptional bursts at dusk. On the other hand, during the repression phase, CLK binding to the promoter region might become less stable and more stochastic, due to the chromatin being more compacted. Consequently, fewer than 50% of the clock neurons exhibited transcriptional bursts, which are also characterized by significantly reduced sizes. Notably, during the activation phase at dusk, we observed a widespread ATAC signal encompassing the entire gene body of Timeless, a phenomenon absent during the repression phase at dawn. The dynamics of transcriptional bursts can be affected by a range of factors, including chromatin accessibility and the proximity between promoters and enhancers.

### How might PER protein drive chromatin compaction during the repression phase?

Past studies have shown that the activators, CLOCK and BMAL1, interact with histone acetlytransferases (HATs) such as p300 and CBP [65], as well as the methyltransferase MLL1 [66], to promote acetylation and methylation of histones, respectively, to promote an open chromatin state to facilitate transcription. Intriguingly, CLOCK has also been reported to have intrinsic HAT activity [67]. On the other hand, PER/CRY complex is known to be a large macromolecular complex composed of at least 25 proteins [68,69]. PER protein has been shown to recruit several transcriptional repressor complexes, such as Mi-2/nucleosome remodelling and deacetylase (NuRD) [70], SIN3-HDAC [71], and Hp1γ–Suv39h histone methyltransferase [72], as well as RNA helicases DDX5 and DHX9 [73], to target genes to promote repression. The interaction of PER with these transcriptional repressor complexes may promote chromatin compaction, essential for the effective silencing of target genes and the creation of rhythmic gene expression patterns vital to circadian regulation. Subsequent studies could delve deeper into the molecular mechanisms controlling chromatin accessibility rhythms within clock neurons.

Chromatin accessibility plays a critical role in regulating gene expression and determining cell fate. As animals develop, distinct DNA regions become variably accessible to transcriptional machinery, ensuring timely and appropriate gene expression at the right stages of development [3,4]. Although chromatin configuration, typically set during development, tends to remain stable throughout the lifespan of the cell [3,4], our research presents divergent findings in clock neurons. Our research uncovers a dynamic chromatin landscape in Drosophila clock neurons: chromatin accessibility of regulatory elements of clock-regulated genes is not static but rather oscillates within a 24-hour day, is dependent on a functional clock, and this rhythmic pattern recurs daily.

## Materials and methods

### Fly stocks

Flies were raised on standard cornmeal/yeast media and maintained at room temperature (20 to 22°C) under a 12h:12h LD schedule. The following flies used in the study were previously described or obtained from the Bloomington Stock Center: *Clk-GAL4* [27], *per01* [26]. Flies were entrained to Light-Dark (LD) cycles where they were exposed to 12-hour Light-Dark (LD) cycles for 5–7 days, followed by a shift to complete darkness (DD) for another 6–7 days. The initiation of the light phase is labeled as Zeitgeber Time (ZT) 0, while ZT12 signifies the beginning of the dark period. When referencing the times in the continuous darkness phase, we use Circadian Time (CT)—with CT24 (subjective dawn) marking the time when lights would have been turned on and CT36 (subjective dusk) the time when lights would have been turned off in second day of darkness (DD2). The labels DD1 and DD2 represent the first and second days of complete darkness, respectively. To make the UAS-GFP-NLS fly lines, coding sequences of GFP-NLS-tetR fusion protein was synthesized and cloned into UAS expression plasmid pJFRC-MUH (Addgene #26213) by GenScript Biotech, and this plasmid was microinjected into phiC31 integrase line with docking site attP40.

### Hybridization Chain Reaction Fluorescence In Situ Hybridization (HCR-FISH)

To perform Hybridization Chain Reaction Fluorescence In Situ Hybridization (HCR-FISH) in whole-mount Drosophila brains, we adapted our previous protocol on single molecule RNA-FISH [74]. Probes and amplifier hairpins were synthesized by Molecular Instruments. In our HCR-FISH protocol, the amplification step was done for ~15 hours, which is slightly longer than the duration recommended by Molecular Instruments Inc. This amplification duration has been determined to be optimal for conducting intron-specific HCR-FISH experiments. Furthermore, due to the compact size of introns, we were limited to designing around five probes per intron. This number is significantly below the preferred "quantitative probe set" size of at least 20 probes. For our HCR-FISH experiments, we housed the flies in density-regulated food vials, each containing 4 females and 4 males. They were acclimated in incubators for 5–7 days. All imaging tests were conducted on male or female flies aged between 5–7 days. Notably, there were no observable differences in the outcomes of our experiments between genders. Specific fly genotypes and Zeitgeber Time (ZT) details are provided in the accompanying Fig legends. To express transgenes in the brain’s clock neurons, we employed the GAL4/UAS system. We captured our images using the Zeiss LSM800 confocal microscope, equipped with an AiryScan super-resolution module that offers 125 nm lateral and 350 nm axial resolution. Imaging was done utilizing a 63x Plan-Apochromat Oil (N.A. 1.4) objective, with laser lines of 405, 488, and 561 nm. Our imaging sessions included collecting Z-stack series (with approximately 250 nm per Z-slice) of distinct clock neurons. Following image acquisition, we imported the CZI files into ImageJ, ensuring we maintained a lossless 16-bit resolution for each channel, using the Bio-Formats Importer to process them as composite images. Subsequently, we manually delineated regions of interest (ROIs) on the channel, fine-tuning the white values to optimize visualization. To guarantee an unbiased fluorescence analysis, we remained consistent with our visualization settings across all images, keeping them specific to each fly line and neuron type. To measure fluorescence intensity of HCR-FISH spot, we used the ImageJ software to measure both the mean pixel brightness (in arbitrary units or a.u.) and the geometric area (in μm^2) of the designated ROI. To calculate the integrated intensity of the spot, we multiplied the pixel brightness with the geometric area, resulting in a numeric value represented in a.u.×μm^2.

### Brain dissociation and flow cytometry

To obtain suspension of clock neurons, we adapted steps described in [29]. Flies were anesthetized and brains were dissected for up to an hour with Schneider’s medium (Sigma S0146) supplemented with 1% bovine serum albumin (BSA, Sigma A7030). Brains were kept on ice, and we aimed to get 60 brains per sample. After dissection, we added collagenase (Sigma C0130) to a final concentration of 2mg/mL. Samples were incubated at 37C for 20min without mixing. We then adjusted solution volume to 200uL and triturated by pipetting with standard 200uL tips 1Hz for up to 80–100 times. Cells were resuspended in 300uL PBS with 0.1% BSA by centrifugation at 300g for 5min at 4C. To obtain a positive control for dead cell marker, suspension equivalent to 3 brains were heated at 60C for 5min. DAPI was added at a final concentration of 2ug/mL as dead cell marker. DAPI at low concentration is cell-impermeable for fly live cells. Finally, suspensions were filtered using FACS tube with strainer cap (Falcon 352235) to obtain single cell suspension and live clock neurons were sorted with FACS sorter (BD Aria III). Cells were sorted into 1.5mL DNA low-bind tubes (Eppendorf) with 50uL PBS with 0.1% BSA. DAPI threshold was set based on the dead cell control sample and GFP threshold was set based on the expected fraction of clock neurons in the whole brain (0.1%). We typically obtained 1500–3000 clock neurons per sample. For non-clock cell control samples, we sorted ten thousand live cells of similar sizes. BSA solution was filtered with 0.22um polyethersulfone membrane (Millipore GPWP047000) and solutions were kept at 4C and reused for up to 5 days to prevent potential contamination. All plastics (including tubes and tips) were coated with PBS 1% BSA for all steps starting trituration to prevent cell loss.

### Assay for transposase-accessible chromatin by sequencing (ATAC-seq)

We adapted steps described in [25,29]. Below, we describe our experiment and analysis procedure.

#### Transposition reaction

Sorted clock neurons were immediately used for ATAC assay after sorting. All plastics were pretreated with 1XPBS with 1% BSA to prevent cell/nucleus loss. First, cells were spun down at 600g for 8min, 4C using spin-out rotor centrifuge. Supernatant was carefully pipetted out and cells were resuspended in 50uL of ice-cold nuclei lysis buffer (10mM Tris-Cl pH 7.4, 3mM MgCl2, 10mM NaCl, 0.1% IGEPAL CA-630) by gently pipetting a few times on ice. Next, nuclei were pelleted at 1200g for 12min, 4°C. Supernatant was carefully pipetted out and permeabilized nuclei were resuspended in 10uL of transposition solution (5uL 2xTD buffer, 0.5uL loaded Tn5 transposase, 4.5uL ultrapure water) by gently pipetting. 2xTD buffer and Tn5 transposase were commercially available (Diagenode C01019043, C01070012). Reactions were incubated at 37C for 30min without agitation, after which DNA was purified by silica column (Zymo DCC-5) with final volume of 20uL using elution buffer. Elution buffer was heated to 55°C and we performed two elution steps (10uL each) to increase yield. Purified DNA was stored at -20°C for up to one week before library preparation.

#### Library preparation and sequencing

Transposed DNA was adjusted to volume of 20uL for each sample and limited-cycle PCR mix was prepared (20uL transposed DNA, 5uL single-indexed Nextera primer mix at 10uM each, 25uL NEBNext High-fidelity PCR master mix). We purchased Nextera primer mix from Integrated DNA technologies which provides universal 8bp index sequences. Next, we performed pre-amplification to repair nicks and then determined amplification cycle numbers by qPCR following standard procedure. We made sure that amplification is in exponential phase and typically 8–10 additional cycles were used to obtain sufficient product for sequencing (>5nM in 20uL after purification). Amplified libraries were purified by non-selective SPRI bead purification and size distribution was assessed by gel electrophoresis (Agilent Tapestation, high-sensitivity DNA 5000 assay). If excess adapter dimers were observed, an extra size-selective SPRI purification was performed. Libraries were then sequenced by shared NovaSeq S4 flow cell, paired-end 150bp cycle targeting 30–50 million reads per sample.

#### Alignment and quality controls

Reads were trimmed to remove sequencing adapters (cutadapt 4.1) and aligned against the dm6 genome assembly using Bowtie2 (version 2.4.5) with parameters ‘—very-sensitive—dovetail -X 1000’. After alignment, mitochondrial reads were removed (samtools 1.15.1) and PCR duplicates were removed using Picard Tools (version 2.27.4). MultiQC (version 1.13a) was used to aggregate result summaries and ATACseqQC (version 1.21.0) was used for standardized quality control designed for ATAC-seq. We typically observed an alignment rate of 30–50%. Unaligned fragments show contamination enriched in bacterial and fungal sequences (NCBI BLAST). Mitochondrial DNA (mtDNA) reads were around 3–5% and PCR duplicate rate was typically around 50%. Overall, we obtained 6–15 million unique non-mtDNA aligned reads.

#### Peak calling and differential analysis

Alignment BAMs were converted to BED with Rsamtools (version 2.13.0) and MACS2 (version 2.2.7.1) was used for peak calling with typical ATAC analysis parameters ‘-f BED -g dm -q 0.001 –nomodel—shift -37—extsize 74—min-length 74—max-gap 74—keep-dup all’. To quantify chromatin accessibility of each peak, we counted read ends that map within the peak using a publicly available R custom package edgeCounter (https://github.com/yeyuan98/edgeCounter). Counts were then used for differential analysis with DESeq2 (version 1.38.3). Pile-up tracks are publicly available on UCSC genome browser (https://tinyurl.com/2ftuhv5p).

### Additional bioinformatics analyses

HOMER (version 4.11) was used for de-novo and known motif enrichment analysis. gProfiler was used for gene ontology enrichment in genes containing differential peaks. Specifically, we used gProfiler2 (g:GOSt module) with g:SCS threshold and unordered query. We provided all accessible genes as the background gene set to reject trivial enrichment results such as “neuron development”. All genes showing ATAC signal were used to scope the statistical domain to remove non-specific enrichment. HOMER de novo analysis results are publicly browsable (https://yeyuan98.github.io/motifAnalysisExports/). Pairwise correlation was computed by counting ATAC reads in 10kb genomic bins. Other analyses shown in this study were performed with packages from the R Bioconductor project [75] including genomic feature annotation using ChIPpeakAnno [76]. The pile-up tracks of all genomic loci from all replicates at all timepoints are readily available on the UCSC genome browser at - **http://tinyurl.com/2ftuhv5p**. To visualize the individual tracks: 1) load the session with the provided URL, 2) click on individual tracks to change their display settings, and 3) set the “Overlay method” to “none”.

Published RNA-seq data of LNv, DN, and LNd [32] was aligned against the FlyBase Release 6.45 assembly using STAR aligner. List of identified rhythmic genes in each cluster from a published single cell RNA-seq data [28] was used to compare our ATAC-seq results to scRNA-seq data.

### Analysis of locomotor activity and rhythmicity

We investigated the locomotor activity of individual adult male flies (3–5 days old) using TriKinetics DAM2 Drosophila Activity Monitors. Each fly was placed in a glass capillary tube (around 4 mm in diameter and 5 cm long) containing food made of 2% agar and 4% sucrose. These monitors, equipped with infrared sensors, detect when the flies move across the tube’s midpoint, causing infrared beam breaks. The monitors were stationed in incubators, and as the flies moved, their activity, measured as infrared beam breaks, was recorded on a connected computer. For behavior analysis, we divided this activity data into 30-minute intervals. To process and visualize this data, we utilized both ClockLab (Actimetrics) software. Each fly’s activity was normalized such that its average daily activity (over 48 intervals) equaled 1. By computing the population mean of this normalized activity, we produced normalized activity plots which are showcased in the Figs. For analyzing rhythmicity, we assessed the activity counts from each fly during the total darkness phase post-entrainment. Using the ClockLab software, we employed a chi-square periodogram analysis (confidence level set at 0.001) to determine the free-running period of the circadian clock and each fly’s rhythmicity. The results from this analysis, the "Power" and "Significance" metrics, enabled us to derive a "Rhythmic Power" value, representing the robustness of each fly’s rhythm.

### Statistical analysis

For assessing the characteristics of HCR-FISH spots, including fluorescence intensity and the percentage of cells with spots, data were aggregated from multiple hemi-brain images. These images were taken across over three separate experiments, ensuring a robust pool of biological replicates. When measuring fluorescence, each neuron was unique to a brain and was measured only once. For behavioral studies, the sample size ranged between 30 to 60 flies, only male flies were used for behavior experiments. For ATAC-seq experiments, we dissected ~60 brains from both male and female flies. We repeated each experiment four times. All data was plotted and statistically analyzed using OriginPro from OriginLab (Northampton, MA, USA).

## Supporting information

S1 Fig Experimental schema for entrainment and FACS procedure. **(A)** Schema of *Drosophila* clock neuron subgroups shown in a hemi-brain. (**B**) Behavior actogram of *Clk-GAL4>UAS-GFP-NLS* flies. These flies were entrained to LD cycles (ZT0: lights on; ZT12: lights off) for 5 d and released into DD for 7 d. Averaged population locomotor-activity profiles of flies (n = 31) in LD and DD with rest-activity shown for two consecutive days in the same line. These flies display rhythmic behaviors with a period of 23.70 ± 0.05 h, with activity peaks around the time of lights on and lights off. (**C**) Representative plots for the fluorescence activated cell sorting procedure. Left panel shows DAPI-forward scatter (FSC) plot for a typical dead-cell control. Right panel shows a representative DAPI-FSC plot for experiment samples. Typical cell viability is 80~90%. (**D**) Representative side scatter (SSC)-GFP plot where live (DAPI-) cells are gated by GFP fluorescence. GFP threshold for clock neurons is set based on ~0.1% expected positive rate. Clock neurons show significantly higher GFP signal, typically >10-fold compared to the GFP-negative population. Green line shows a representative GFP-gate setting. (**E**) Representative TSS enrichment score distribution showing clear signal enrichment immediately upstream of TSS. Enrichment score is computed using the ATACseqQC package.
(DOCX)

S2 Fig ATAC signal pile-up tracks for core clock genes in GFP-positive clock neurons and GFP-negative non-clock cells. (**A**) ATAC signal pile-up tracks at *per*, *vri*, *Pdp1* and *cyc* loci in clock neurons (GFP-positive) and non-clock cells (GFP-negative). We did not observe any accessibility changes in the *cycle* locus. Differentially accessible peaks are marked in black boxes. (**B-D**) ATAC signal pile-up tracks at the tim locus (B), per locus (C), andneuropeptide CCHa1 locus (D) showing individual biological replicates.
(DOCX)

S3 Fig Schema of ATAC signal differential analysis. (**A**) Illustration of how we counted ATAC signal from MACS2 called peaks using the custom edgeCounter package (see Methods). (**B,C**) Binary heatmap comparing MACS2 called peaks under LD and DD conditions (B) and differentially accessible peaks at dawn, dusk, subjective dawn, and subjective dusk (C). (**D**) Distribution of subjective dusk and subjective dawn-accessible peaks with respect to known genomic features reported by ChIPpeakAnno. (**E**) Quantile-quantile plots showing correlation between ZT and CT peaks at dawn (ZT0/CT24) and dusk (ZT12/CT36). Differential peaks that are conserved between ZT and CT conditions (y-axis) shows lower adjusted p-values compared to peaks that are not conserved (i.e., only found in ZT, x-axis).
(DOCX)

S4 Fig ATAC signal pile-up tracks of core clock genes *Clk*, *Pdp1 and vri* under LD and DD conditions. (**A,B**) *Clk* locus is more accessible at dawn (ZT0/CT24) compared to dusk while *per* locus shows the opposite pattern. (**C**) Transcript variants of Pdp1 exhibit varying accessibility changes. The "long variants" possess a regulatory element that is more accessible at dusk, whereas the "short variants" display the opposite pattern. (**D**) *vri* locus also shows complex differential peak patterns. It possesses regulatory elements that are more accessible at dawn and dusk in GFP-positive clock neurons. Additionally, *vri* locus also possesses a regulatory element that is more accessible specifically at dusk even in non-clock cells (GFP-negative). Fold change and adjusted p-value are shown at the bottom of each of the differential peaks identified.
(DOCX)

S5 Fig Genes with peaks that are more accessible at both dusk and dawn. (**A**) List of genes that have differentially accessible peaks at both dusk and dawn. In all, 11 genes meet this criterion, and only those that exhibit cyclical changes in mRNA levels are presented here (6 out of 11). (**B**) Expression level of “short” and “long” transcript variants of Pdp1 in differential clock neuron subgroups (DN1, LNv, LNd) and a non-clock cell control (dopamine neurons, TH). While short variants show robust cycling profiles in all clock neuron groups, long variants are not cycling in DN1 neurons. Analysis was performed with publicly available data [28].
(DOCX)

S6 Fig Overlap analysis of differential ATAC peaks to CLK-binding regions While some differential ATAC peaks overlap with CLK-binding regions identified in a previous microarray study [42], most (>90%) differential ATAC peaks are far away from CLK-binding regions with a median distance of ~30kb. (DOCX)

S7 Fig Transcriptional activity of *Shaker* in non-clock neurons. (**A**) Representative images of *Shaker* intron 4 HCR-FISH in non-clock cells (*fru*-positive neurons). B) Quantification of fluorescence intensity of Shaker intron spot in *fru*-positive neurons. The statistical test used was a two-sided Student’s t-test, no significance was detected. Scale bar: 2μm.
(DOCX)

S1 Table Analysis of pervasive gene accessibility genome-wide. (XLSX)

S2 Table Annnotations of all the differential peaks identified in ZT and CT conditions. (XLSX)

## References

[1] Flavahan WA, Gaskell E, Bernstein BE. Epigenetic plasticity and the hallmarks of cancer. Science. 2017;357(6348). doi: 10.1126/science.aal2380 ; PubMed Central PMCID: PMC5940341. 28729483 PMC5940341

[2] Janssens J, Aibar S, Taskiran II, Ismail JN, Gomez AE, Aughey G, et al. Decoding gene regulation in the fly brain. Nature. 2022;601(7894):630–6. Epub 20220105. doi: 10.1038/s41586-021-04262-z . 34987221

[3] Buenrostro JD, Corces MR, Lareau CA, Wu B, Schep AN, Aryee MJ, et al. Integrated Single-Cell Analysis Maps the Continuous Regulatory Landscape of Human Hematopoietic Differentiation. Cell. 2018;173(6):1535–48 e16. Epub 20180426. doi: 10.1016/j.cell.2018.03.074 ; PubMed Central PMCID: PMC5989727. 29706549 PMC5989727

[4] Klemm SL, Shipony Z, Greenleaf WJ. Chromatin accessibility and the regulatory epigenome. Nat Rev Genet. 2019;20(4):207–20. doi: 10.1038/s41576-018-0089-8 . 30675018

[5] Ho L, Miller EL, Ronan JL, Ho WQ, Jothi R, Crabtree GR. esBAF facilitates pluripotency by conditioning the genome for LIF/STAT3 signalling and by regulating polycomb function. Nat Cell Biol. 2011;13(8):903–13. Epub 20110724. doi: 10.1038/ncb2285 ; PubMed Central PMCID: PMC3155811. 21785422 PMC3155811

[6] Bell-Pedersen D, Cassone VM, Earnest DJ, Golden SS, Hardin PE, Thomas TL, et al. Circadian rhythms from multiple oscillators: lessons from diverse organisms. Nat Rev Genet. 2005;6(7):544–56. Epub 2005/06/14. doi: 10.1038/nrg1633 ; PubMed Central PMCID: PMC2735866. 15951747 PMC2735866

[7] Patke A, Young MW, Axelrod S. Molecular mechanisms and physiological importance of circadian rhythms. Nat Rev Mol Cell Bio. 2020;21(2):67–84. doi: 10.1038/s41580-019-0179-2 WOS:000510887000006. 31768006

[8] Takahashi JS. Transcriptional architecture of the mammalian circadian clock. Nat Rev Genet. 2017;18(3):164–79. Epub 20161219. doi: 10.1038/nrg.2016.150 ; PubMed Central PMCID: PMC5501165. 27990019 PMC5501165

[9] Mermet J, Yeung J, Hurni C, Mauvoisin D, Gustafson K, Jouffe C, et al. Clock-dependent chromatin topology modulates circadian transcription and behavior. Genes Dev. 2018;32(5–6):347–58. Epub 20180323. doi: 10.1101/gad.312397.118 ; PubMed Central PMCID: PMC5900709. 29572261 PMC5900709

[10] Kim YH, Marhon SA, Zhang Y, Steger DJ, Won KJ, Lazar MA. Rev-erbalpha dynamically modulates chromatin looping to control circadian gene transcription. Science. 2018;359(6381):1274–7. Epub 20180208. doi: 10.1126/science.aao6891 ; PubMed Central PMCID: PMC5995144. 29439026 PMC5995144

[11] Xiao Y, Yuan Y, Jimenez M, Soni N, Yadlapalli S. Clock proteins regulate spatiotemporal organization of clock genes to control circadian rhythms. Proc Natl Acad Sci U S A. 2021;118(28). doi: 10.1073/pnas.2019756118 ; PubMed Central PMCID: PMC8285898. 34234015 PMC8285898

[12] Zhao H, Sifakis EG, Sumida N, Millan-Arino L, Scholz BA, Svensson JP, et al. PARP1- and CTCF-Mediated Interactions between Active and Repressed Chromatin at the Lamina Promote Oscillating Transcription. Mol Cell. 2015;59(6):984–97. Epub 20150827. doi: 10.1016/j.molcel.2015.07.019 . 26321255

[13] Belden WJ, Loros JJ, Dunlap JC. Execution of the circadian negative feedback loop in Neurospora requires the ATP-dependent chromatin-remodeling enzyme CLOCKSWITCH. Mol Cell. 2007;25(4):587–600. doi: 10.1016/j.molcel.2007.01.010 . 17317630

[14] Cha J, Zhou M, Liu Y. CATP is a critical component of the Neurospora circadian clock by regulating the nucleosome occupancy rhythm at the frequency locus. EMBO Rep. 2013;14(10):923–30. Epub 20130820. doi: 10.1038/embor.2013.131 ; PubMed Central PMCID: PMC3807232. 23958634 PMC3807232

[15] Wang B, Kettenbach AN, Gerber SA, Loros JJ, Dunlap JC. Neurospora WC-1 recruits SWI/SNF to remodel frequency and initiate a circadian cycle. PLoS Genet. 2014;10(9):e1004599. Epub 20140925. doi: 10.1371/journal.pgen.1004599 ; PubMed Central PMCID: PMC4177678. 25254987 PMC4177678

[16] Kwok RS, Li YH, Lei AJ, Edery I, Chiu JC. The Catalytic and Non-catalytic Functions of the Brahma Chromatin-Remodeling Protein Collaborate to Fine-Tune Circadian Transcription in Drosophila. PLoS Genet. 2015;11(7):e1005307. Epub 20150701. doi: 10.1371/journal.pgen.1005307 ; PubMed Central PMCID: PMC4488936. 26132408 PMC4488936

[17] Lugena AB, Zhang Y, Menet JS, Merlin C. Genome-wide discovery of the daily transcriptome, DNA regulatory elements and transcription factor occupancy in the monarch butterfly brain. PLoS Genet. 2019;15(7):e1008265. Epub 20190723. doi: 10.1371/journal.pgen.1008265 ; PubMed Central PMCID: PMC6677324. 31335862 PMC6677324

[18] Weizman EN, Tannenbaum M, Tarrant AM, Hakim O, Levy O. Chromatin dynamics enable transcriptional rhythms in the cnidarian Nematostella vectensis. PLoS Genet. 2019;15(11):e1008397. Epub 20191106. doi: 10.1371/journal.pgen.1008397 ; PubMed Central PMCID: PMC6834241. 31693674 PMC6834241

[19] Zhang Y, Chen G, Deng L, Gao B, Yang J, Ding C, et al. Integrated 3D genome, epigenome and transcriptome analyses reveal transcriptional coordination of circadian rhythm in rice. Nucleic Acids Res. 2023;51(17):9001–18. doi: 10.1093/nar/gkad658 ; PubMed Central PMCID: PMC10516653. 37572350 PMC10516653

[20] Dubowy C, Sehgal A. Circadian Rhythms and Sleep in Drosophila melanogaster. Genetics. 2017;205(4):1373–97. doi: 10.1534/genetics.115.185157 ; PubMed Central PMCID: PMC5378101. 28360128 PMC5378101

[21] Nitabach MN, Taghert PH. Organization of the Drosophila circadian control circuit. Curr Biol. 2008;18(2):R84–93. doi: 10.1016/j.cub.2007.11.061 . 18211849

[22] Cyran SA, Buchsbaum AM, Reddy KL, Lin MC, Glossop NR, Hardin PE, et al. vrille, Pdp1, and dClock form a second feedback loop in the Drosophila circadian clock. Cell. 2003;112(3):329–41. doi: 10.1016/s0092-8674(03)00074-6 . 12581523

[23] Gunawardhana KL, Hardin PE. VRILLE Controls PDF Neuropeptide Accumulation and Arborization Rhythms in Small Ventrolateral Neurons to Drive Rhythmic Behavior in Drosophila. Curr Biol. 2017;27(22):3442–53 e4. Epub 20171102. doi: 10.1016/j.cub.2017.10.010 . 29103936

[24] Benito J, Zheng H, Hardin PE. PDP1epsilon functions downstream of the circadian oscillator to mediate behavioral rhythms. J Neurosci. 2007;27(10):2539–47. doi: 10.1523/JNEUROSCI.4870-06.2007 ; PubMed Central PMCID: PMC1828026. 17344391 PMC1828026

[25] Buenrostro JD, Giresi PG, Zaba LC, Chang HY, Greenleaf WJ. Transposition of native chromatin for fast and sensitive epigenomic profiling of open chromatin, DNA-binding proteins and nucleosome position. Nat Methods. 2013;10(12):1213–8. Epub 20131006. doi: 10.1038/nmeth.2688 ; PubMed Central PMCID: PMC3959825. 24097267 PMC3959825

[26] Konopka RJ, Benzer S. Clock mutants of Drosophila melanogaster. Proc Natl Acad Sci U S A. 1971;68(9):2112–6. doi: 10.1073/pnas.68.9.2112 ; PubMed Central PMCID: PMC389363. 5002428 PMC389363

[27] Gummadova JO, Coutts GA, Glossop NR. Analysis of the Drosophila Clock promoter reveals heterogeneity in expression between subgroups of central oscillator cells and identifies a novel enhancer region. J Biol Rhythms. 2009;24(5):353–67. doi: 10.1177/0748730409343890 . 19755581

[28] Ma D, Przybylski D, Abruzzi KC, Schlichting M, Li Q, Long X, et al. A transcriptomic taxonomy of Drosophila circadian neurons around the clock. Elife. 2021;10. Epub 20210113. doi: 10.7554/eLife.63056 ; PubMed Central PMCID: PMC7837698. 33438579 PMC7837698

[29] Brovkina MV, Duffie R, Burtis AEC, Clowney EJ. Fruitless decommissions regulatory elements to implement cell-type-specific neuronal masculinization. PLoS Genet. 2021;17(2):e1009338. Epub 20210218. doi: 10.1371/journal.pgen.1009338 ; PubMed Central PMCID: PMC7924761. 33600447 PMC7924761

[30] Zhang Y, Liu T, Meyer CA, Eeckhoute J, Johnson DS, Bernstein BE, et al. Model-based analysis of ChIP-Seq (MACS). Genome Biol. 2008;9(9):R137. Epub 20080917. doi: 10.1186/gb-2008-9-9-r137 ; PubMed Central PMCID: PMC2592715. 18798982 PMC2592715

[31] Love MI, Huber W, Anders S. Moderated estimation of fold change and dispersion for RNA-seq data with DESeq2. Genome Biol. 2014;15(12):550. doi: 10.1186/s13059-014-0550-8 ; PubMed Central PMCID: PMC4302049. 25516281 PMC4302049

[32] Abruzzi KC, Zadina A, Luo W, Wiyanto E, Rahman R, Guo F, et al. RNA-seq analysis of Drosophila clock and non-clock neurons reveals neuron-specific cycling and novel candidate neuropeptides. PLoS Genet. 2017;13(2):e1006613. Epub 20170209. doi: 10.1371/journal.pgen.1006613 ; PubMed Central PMCID: PMC5325595. 28182648 PMC5325595

[33] Thurman RE, Rynes E, Humbert R, Vierstra J, Maurano MT, Haugen E, et al. The accessible chromatin landscape of the human genome. Nature. 2012;489(7414):75–82. doi: 10.1038/nature11232 ; PubMed Central PMCID: PMC3721348. 22955617 PMC3721348

[34] Taylor P, Hardin PE. Rhythmic E-box binding by CLK-CYC controls daily cycles in per and tim transcription and chromatin modifications. Mol Cell Biol. 2008;28(14):4642–52. Epub 20080512. doi: 10.1128/MCB.01612-07 ; PubMed Central PMCID: PMC2447118. 18474612 PMC2447118

[35] Bi S, Yue S, Zhang S. Hybridization chain reaction: a versatile molecular tool for biosensing, bioimaging, and biomedicine. Chem Soc Rev. 2017;46(14):4281–98. doi: 10.1039/c7cs00055c . 28573275

[36] Heinz S, Benner C, Spann N, Bertolino E, Lin YC, Laslo P, et al. Simple combinations of lineage-determining transcription factors prime cis-regulatory elements required for macrophage and B cell identities. Mol Cell. 2010;38(4):576–89. doi: 10.1016/j.molcel.2010.05.004 ; PubMed Central PMCID: PMC2898526. 20513432 PMC2898526

[37] Hao H, Allen DL, Hardin PE. A circadian enhancer mediates PER-dependent mRNA cycling in Drosophila melanogaster. Mol Cell Biol. 1997;17(7):3687–93. doi: 10.1128/MCB.17.7.3687 ; PubMed Central PMCID: PMC232220. 9199302 PMC232220

[38] Yu W, Zheng H, Houl JH, Dauwalder B, Hardin PE. PER-dependent rhythms in CLK phosphorylation and E-box binding regulate circadian transcription. Genes Dev. 2006;20(6):723–33. doi: 10.1101/gad.1404406 ; PubMed Central PMCID: PMC1434787. 16543224 PMC1434787

[39] Marcheva B, Perelis M, Weidemann BJ, Taguchi A, Lin H, Omura C, et al. A role for alternative splicing in circadian control of exocytosis and glucose homeostasis. Genes Dev. 2020;34(15–16):1089–105. Epub 20200702. doi: 10.1101/gad.338178.120 ; PubMed Central PMCID: PMC7397853. 32616519 PMC7397853

[40] Maillo C, Martin J, Sebastian D, Hernandez-Alvarez M, Garcia-Rocha M, Reina O, et al. Circadian- and UPR-dependent control of CPEB4 mediates a translational response to counteract hepatic steatosis under ER stress. Nat Cell Biol. 2017;19(2):94–105. Epub 20170116. doi: 10.1038/ncb3461 . 28092655

[41] Cheng AH, Bouchard-Cannon P, Hegazi S, Lowden C, Fung SW, Chiang CK, et al. SOX2-Dependent Transcription in Clock Neurons Promotes the Robustness of the Central Circadian Pacemaker. Cell Rep. 2019;26(12):3191–202 e8. doi: 10.1016/j.celrep.2019.02.068 . 30893593

[42] Abruzzi KC, Rodriguez J, Menet JS, Desrochers J, Zadina A, Luo W, et al. Drosophila CLOCK target gene characterization: implications for circadian tissue-specific gene expression. Genes Dev. 2011;25(22):2374–86. doi: 10.1101/gad.178079.111 ; PubMed Central PMCID: PMC3222903. 22085964 PMC3222903

[43] Rivera J, Keranen SVE, Gallo SM, Halfon MS. REDfly: the transcriptional regulatory element database for Drosophila. Nucleic Acids Res. 2019;47(D1):D828–D34. doi: 10.1093/nar/gky957 ; PubMed Central PMCID: PMC6323911. 30329093 PMC6323911

[44] Negre N, Brown CD, Ma L, Bristow CA, Miller SW, Wagner U, et al. A cis-regulatory map of the Drosophila genome. Nature. 2011;471(7339):527–31. doi: 10.1038/nature09990 ; PubMed Central PMCID: PMC3179250. 21430782 PMC3179250

[45] Bozek M, Cortini R, Storti AE, Unnerstall U, Gaul U, Gompel N. ATAC-seq reveals regional differences in enhancer accessibility during the establishment of spatial coordinates in the Drosophila blastoderm. Genome Res. 2019;29(5):771–83. Epub 20190408. doi: 10.1101/gr.242362.118 ; PubMed Central PMCID: PMC6499308. 30962180 PMC6499308

[46] Celniker SE, Dillon LA, Gerstein MB, Gunsalus KC, Henikoff S, Karpen GH, et al. Unlocking the secrets of the genome. Nature. 2009;459(7249):927–30. doi: 10.1038/459927a ; PubMed Central PMCID: PMC2843545. 19536255 PMC2843545

[47] Koike N, Yoo SH, Huang HC, Kumar V, Lee C, Kim TK, et al. Transcriptional architecture and chromatin landscape of the core circadian clock in mammals. Science. 2012;338(6105):349–54. Epub 20120830. doi: 10.1126/science.1226339 ; PubMed Central PMCID: PMC3694775. 22936566 PMC3694775

[48] Robles MS, Cox J, Mann M. In-vivo quantitative proteomics reveals a key contribution of post-transcriptional mechanisms to the circadian regulation of liver metabolism. PLoS Genet. 2014;10(1):e1004047. Epub 20140102. doi: 10.1371/journal.pgen.1004047 ; PubMed Central PMCID: PMC3879213. 24391516 PMC3879213

[49] Fernandez MP, Berni J, Ceriani MF. Circadian remodeling of neuronal circuits involved in rhythmic behavior. PLoS Biol. 2008;6(3):e69. doi: 10.1371/journal.pbio.0060069 ; PubMed Central PMCID: PMC2270325. 18366255 PMC2270325

[50] Bae K, Lee C, Sidote D, Chuang KY, Edery I. Circadian regulation of a Drosophila homolog of the mammalian Clock gene: PER and TIM function as positive regulators. Mol Cell Biol. 1998;18(10):6142–51. doi: 10.1128/MCB.18.10.6142 ; PubMed Central PMCID: PMC109200. 9742131 PMC109200

[51] Bruning F, Noya SB, Bange T, Koutsouli S, Rudolph JD, Tyagarajan SK, et al. Sleep-wake cycles drive daily dynamics of synaptic phosphorylation. Science. 2019;366(6462). doi: 10.1126/science.aav3617 . 31601740

[52] George H, Terracol R. The vrille gene of Drosophila is a maternal enhancer of decapentaplegic and encodes a new member of the bZIP family of transcription factors. Genetics. 1997;146(4):1345–63. doi: 10.1093/genetics/146.4.1345 ; PubMed Central PMCID: PMC1208080. 9258679 PMC1208080

[53] Blau J, Young MW. Cycling vrille expression is required for a functional Drosophila clock. Cell. 1999;99(6):661–71. doi: 10.1016/s0092-8674(00)81554-8 . 10612401

[54] Shulga YV, Topham MK, Epand RM. Regulation and functions of diacylglycerol kinases. Chem Rev. 2011;111(10):6186–208. Epub 20110729. doi: 10.1021/cr1004106 . 21800853

[55] Cirelli C, Bushey D, Hill S, Huber R, Kreber R, Ganetzky B, et al. Reduced sleep in Drosophila Shaker mutants. Nature. 2005;434(7037):1087–92. doi: 10.1038/nature03486 . 15858564

[56] Wu MN, Joiner WJ, Dean T, Yue Z, Smith CJ, Chen D, et al. SLEEPLESS, a Ly-6/neurotoxin family member, regulates the levels, localization and activity of Shaker. Nat Neurosci. 2010;13(1):69–75. Epub 20091213. doi: 10.1038/nn.2454 ; PubMed Central PMCID: PMC2842941. 20010822 PMC2842941

[57] Li Y, Haynes P, Zhang SL, Yue Z, Sehgal A. Ecdysone acts through cortex glia to regulate sleep in Drosophila. Elife. 2023;12. Epub 20230131. doi: 10.7554/eLife.81723 ; PubMed Central PMCID: PMC9928426. 36719183 PMC9928426

[58] Kamb A, Iverson LE, Tanouye MA. Molecular characterization of Shaker, a Drosophila gene that encodes a potassium channel. Cell. 1987;50(3):405–13. doi: 10.1016/0092-8674(87)90494-6 . 2440582

[59] Kumar S, Chen D, Jang C, Nall A, Zheng X, Sehgal A. An ecdysone-responsive nuclear receptor regulates circadian rhythms in Drosophila. Nat Commun. 2014;5:5697. Epub 20141216. doi: 10.1038/ncomms6697 ; PubMed Central PMCID: PMC4269253. 25511299 PMC4269253

[60] Jaumouille E, Machado Almeida P, Stahli P, Koch R, Nagoshi E. Transcriptional regulation via nuclear receptor crosstalk required for the Drosophila circadian clock. Curr Biol. 2015;25(11):1502–8. Epub 20150521. doi: 10.1016/j.cub.2015.04.017 ; PubMed Central PMCID: PMC4454776. 26004759 PMC4454776

[61] Qu M, Qu H, Jia Z, Kay SA. HNF4A defines tissue-specific circadian rhythms by beaconing BMAL1::CLOCK chromatin binding and shaping the rhythmic chromatin landscape. Nat Commun. 2021;12(1):6350. Epub 20211103. doi: 10.1038/s41467-021-26567-3 ; PubMed Central PMCID: PMC8566521. 34732735 PMC8566521

[62] Zhang R, Lahens NF, Ballance HI, Hughes ME, Hogenesch JB. A circadian gene expression atlas in mammals: implications for biology and medicine. Proc Natl Acad Sci U S A. 2014;111(45):16219–24. Epub 20141027. doi: 10.1073/pnas.1408886111 ; PubMed Central PMCID: PMC4234565. 25349387 PMC4234565

[63] Hughes ME, Grant GR, Paquin C, Qian J, Nitabach MN. Deep sequencing the circadian and diurnal transcriptome of Drosophila brain. Genome Res. 2012;22(7):1266–81. Epub 20120403. doi: 10.1101/gr.128876.111 ; PubMed Central PMCID: PMC3396368. 22472103 PMC3396368

[64] Larson DR, Zenklusen D, Wu B, Chao JA, Singer RH. Real-time observation of transcription initiation and elongation on an endogenous yeast gene. Science. 2011;332(6028):475–8. doi: 10.1126/science.1202142 ; PubMed Central PMCID: PMC3152976. 21512033 PMC3152976

[65] Etchegaray JP, Lee C, Wade PA, Reppert SM. Rhythmic histone acetylation underlies transcription in the mammalian circadian clock. Nature. 2003;421(6919):177–82. Epub 20021211. doi: 10.1038/nature01314 . 12483227

[66] Katada S, Sassone-Corsi P. The histone methyltransferase MLL1 permits the oscillation of circadian gene expression. Nat Struct Mol Biol. 2010;17(12):1414–21. Epub 20101128. doi: 10.1038/nsmb.1961 ; PubMed Central PMCID: PMC6501791. 21113167 PMC6501791

[67] Doi M, Hirayama J, Sassone-Corsi P. Circadian regulator CLOCK is a histone acetyltransferase. Cell. 2006;125(3):497–508. doi: 10.1016/j.cell.2006.03.033 . 16678094

[68] Aryal RP, Kwak PB, Tamayo AG, Gebert M, Chiu PL, Walz T, et al. Macromolecular Assemblies of the Mammalian Circadian Clock. Mol Cell. 2017;67(5):770–82 e6. doi: 10.1016/j.molcel.2017.07.017 ; PubMed Central PMCID: PMC5679067. 28886335 PMC5679067

[69] Cao X, Wang L, Selby CP, Lindsey-Boltz LA, Sancar A. Analysis of mammalian circadian clock protein complexes over a circadian cycle. J Biol Chem. 2023;299(3):102929. Epub 20230120. doi: 10.1016/j.jbc.2023.102929 ; PubMed Central PMCID: PMC9950529. 36682495 PMC9950529

[70] Kim JY, Kwak PB, Weitz CJ. Specificity in circadian clock feedback from targeted reconstitution of the NuRD corepressor. Mol Cell. 2014;56(6):738–48. Epub 20141120. doi: 10.1016/j.molcel.2014.10.017 . 25453762

[71] Duong HA, Robles MS, Knutti D, Weitz CJ. A molecular mechanism for circadian clock negative feedback. Science. 2011;332(6036):1436–9. doi: 10.1126/science.1196766 ; PubMed Central PMCID: PMC3859310. 21680841 PMC3859310

[72] Duong HA, Weitz CJ. Temporal orchestration of repressive chromatin modifiers by circadian clock Period complexes. Nat Struct Mol Biol. 2014;21(2):126–32. Epub 20140112. doi: 10.1038/nsmb.2746 ; PubMed Central PMCID: PMC4227600. 24413057 PMC4227600

[73] Padmanabhan K, Robles MS, Westerling T, Weitz CJ. Feedback regulation of transcriptional termination by the mammalian circadian clock PERIOD complex. Science. 2012;337(6094):599–602. Epub 20120705. doi: 10.1126/science.1221592 . 22767893

[74] Yuan Y, Padilla MA, Clark D, Yadlapalli S. Streamlined single-molecule RNA-FISH of core clock mRNAs in clock neurons in whole mount Drosophila brains. Front Physiol. 2022;13:1051544. Epub 20221109. doi: 10.3389/fphys.2022.1051544 ; PubMed Central PMCID: PMC9682093. 36439243 PMC9682093

[75] Ou J, Liu H, Yu J, Kelliher MA, Castilla LH, Lawson ND, et al. ATACseqQC: a Bioconductor package for post-alignment quality assessment of ATAC-seq data. BMC Genomics. 2018;19(1):169. Epub 20180301. doi: 10.1186/s12864-018-4559-3 ; PubMed Central PMCID: PMC5831847. 29490630 PMC5831847

[76] Zhu LJ, Gazin C, Lawson ND, Pages H, Lin SM, Lapointe DS, et al. ChIPpeakAnno: a Bioconductor package to annotate ChIP-seq and ChIP-chip data. BMC Bioinformatics. 2010;11:237. Epub 20100511. doi: 10.1186/1471-2105-11-237 ; PubMed Central PMCID: PMC3098059. 20459804 PMC3098059

