# Supplementary material for: Clock-dependent chromatin accessibility rhythms regulate circadian transcription
Source: PLoS Genet. 2024 May 28;20(5):e1011278. doi: 10.1371/journal.pgen.1011278 (PMC11161047; doi:10.1371/journal.pgen.1011278)
Supplement: S1 Fig — (A) Schema of Drosophila clock neuron subgroups shown in a hemi-brain. (B) Behavior actogram of Clk-GAL4>UAS-GFP-NLS flies. These flies were entrained to LD cycles (ZT0: lights on; ZT12: lights off) for 5 d and released into DD for 7 d. Averaged population locomotor-activity profiles of flies (n = 31) in LD and DD with rest-activity shown for two consecutive days in the same line. These flies display rhythmic behaviors with a period of 23.70 ± 0.05 h, with activity peaks around the time of lights on and lights off. (C) Representative plots for the fluorescence activated cell sorting procedure. Left panel shows DAPI-forward scatter (FSC) plot for a typical dead-cell control. Right panel shows a representative DAPI-FSC plot for experiment samples. Typical cell viability is 80~90%. (D) Representative side scatter (SSC)-GFP plot where live (DAPI-) cells are gated by GFP fluorescence. GFP threshold for clock neurons is set based on ~0.1% expected positive rate. Clock neurons show significantly higher GFP signal, typically >10-fold compared to the GFP-negative population. Green line shows a representative GFP-gate setting. (E) Representative TSS enrichment score distribution showing clear signal enrichment immediately upstream of TSS. Enrichment score is computed using the ATACseqQC package. (DOCX) [file pgen.1011278.s001.docx]

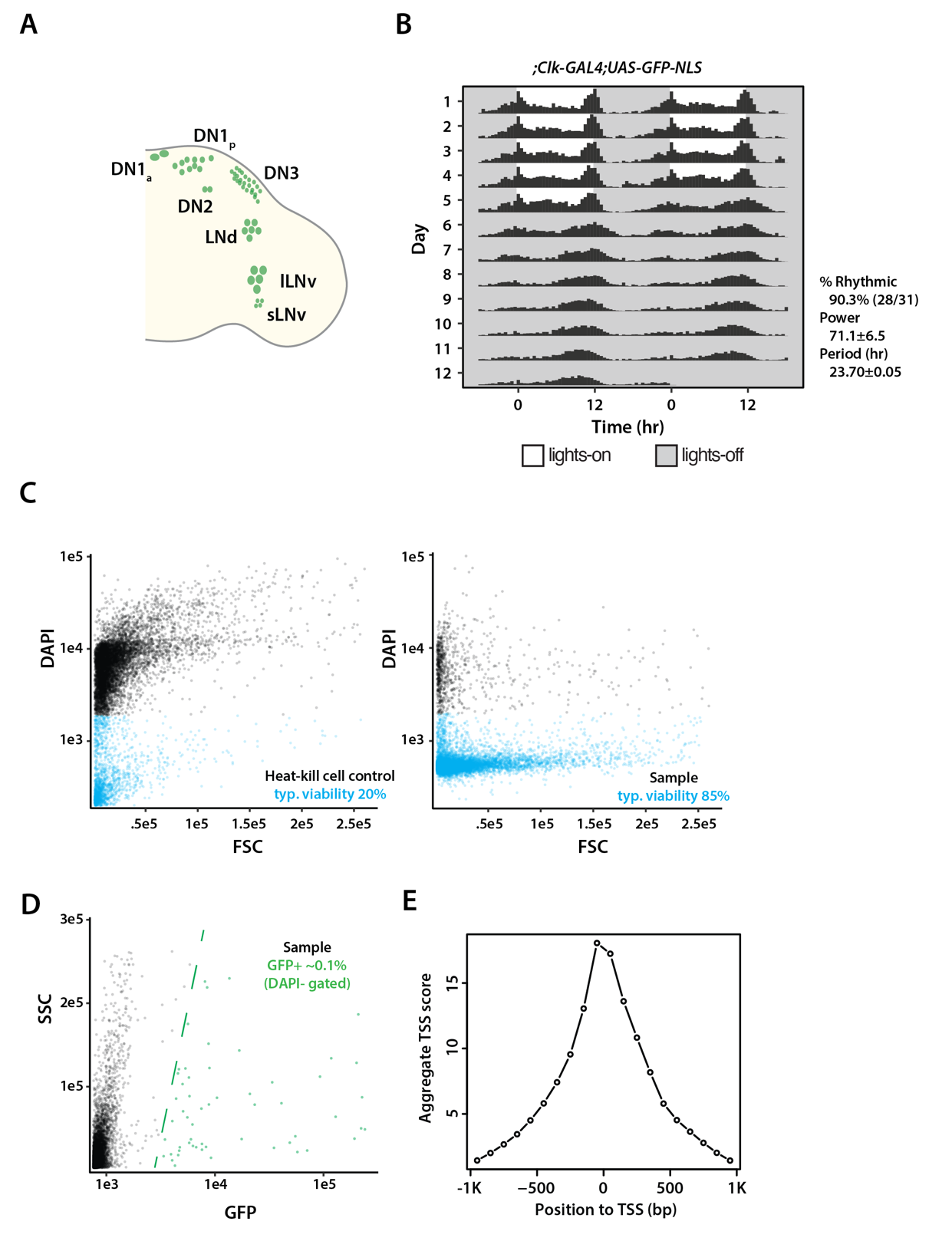


**S1 Fig. Experimental schema for entrainment and FACS procedure. (A)** Schema of *Drosophila* clock neuron subgroups shown in a hemi-brain. (**B**) Behavior actogram of *Clk-GAL4>UAS-GFP-NLS* flies. These flies were entrained to LD cycles (ZT0: lights on; ZT12: lights off) for 5 d and released into DD for 7 d. Averaged population locomotor-activity profiles of flies (n = 31) in LD and DD with rest-activity shown for two consecutive days in the same line. These flies display rhythmic behaviors with a period of 23.70 ± 0.05 h, with activity peaks around the time of lights on and lights off. (**C**) Representative plots for the fluorescence activated cell sorting procedure. Left panel shows DAPI-forward scatter (FSC) plot for a typical dead-cell control. Right panel shows a representative DAPI-FSC plot for experiment samples. Typical cell viability is $80\sim90\%$. (**D**) Representative side scatter (SSC)-GFP plot where live (DAPI-) cells are gated by GFP fluorescence. GFP threshold for clock neurons is set based on $\sim0.1\%$ expected positive rate. Clock neurons show significantly higher GFP signal, typically >10-fold compared to the GFP-negative population. Green line shows a representative GFP-gate setting. (**E**) Representative TSS enrichment score distribution showing clear signal enrichment immediately upstream of TSS. Enrichment score is computed using the ATACseqQC package.
