# Supplementary material for: Clock-dependent chromatin accessibility rhythms regulate circadian transcription
Source: PLoS Genet. 2024 May 28;20(5):e1011278. doi: 10.1371/journal.pgen.1011278 (PMC11161047; doi:10.1371/journal.pgen.1011278)
Supplement: S2 Fig — (A) ATAC signal pile-up tracks at per, vri, Pdp1 and cyc loci in clock neurons (GFP-positive) and non-clock cells (GFP-negative). We did not observe any accessibility changes in the cycle locus. Differentially accessible peaks are marked in black boxes. (B-D) ATAC signal pile-up tracks at the tim locus (B), per locus (C), andneuropeptide CCHa1 locus (D) showing individual biological replicates. (DOCX) [file pgen.1011278.s002.docx]

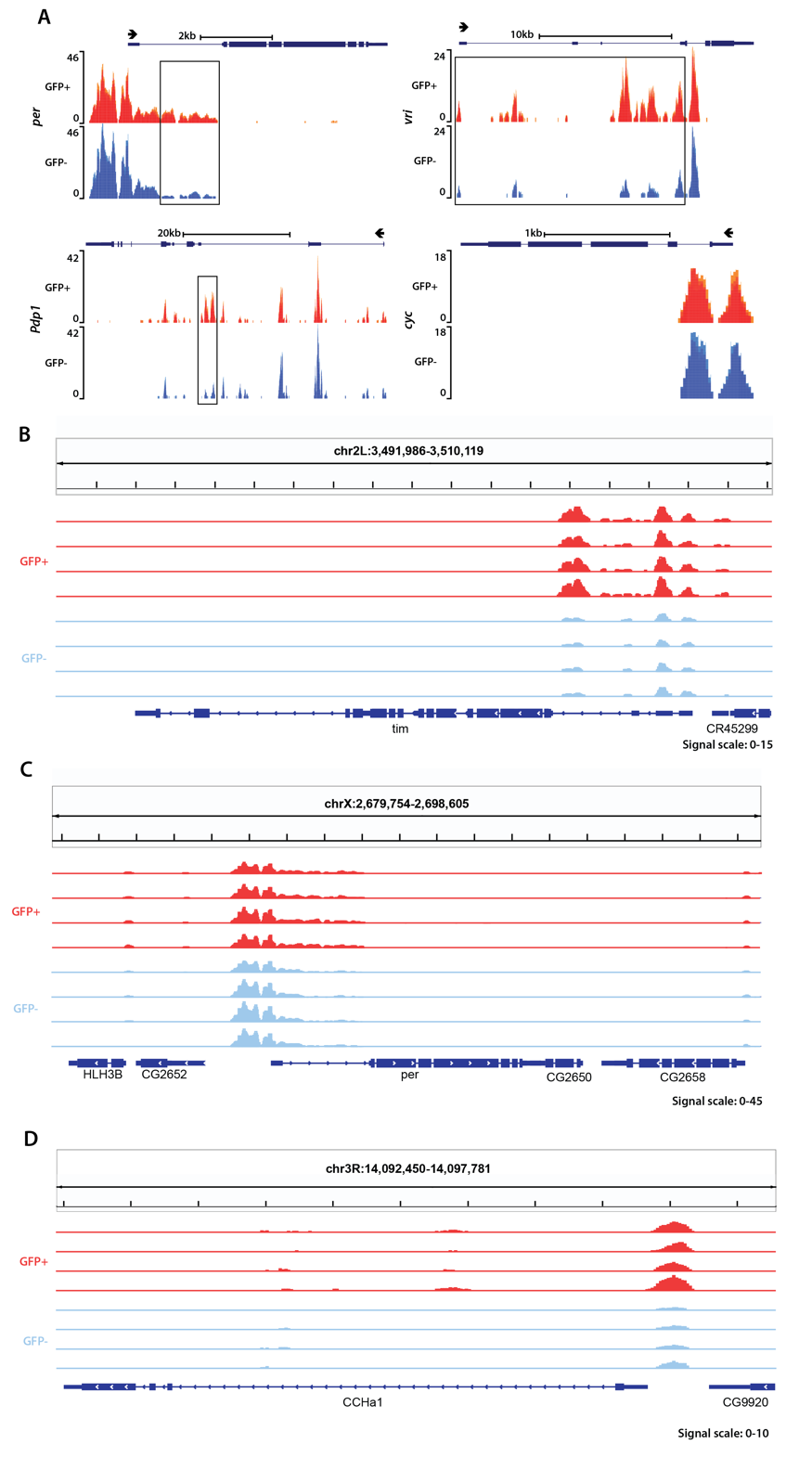


**S2 Fig. ATAC signal pile-up tracks for core clock genes in GFP-positive clock neurons and GFP-negative non-clock cells.**

(**A**) ATAC signal pile-up tracks at *per, vri, Pdp1* and *cyc* loci in clock neurons (GFP-positive) and non-clock cells (GFP-negative). We did not observe any accessibility changes in the *cycle* locus. Differentially accessible peaks are marked in black boxes. (**B-D**) ATAC signal pile-up tracks at the tim locus (B), per locus (C), andneuropeptide CCHa1 locus (D) showing individual biological replicates.
