# Supplementary material for: Clock-dependent chromatin accessibility rhythms regulate circadian transcription
Source: PLoS Genet. 2024 May 28;20(5):e1011278. doi: 10.1371/journal.pgen.1011278 (PMC11161047; doi:10.1371/journal.pgen.1011278)
Supplement: S3 Fig — (A) Illustration of how we counted ATAC signal from MACS2 called peaks using the custom edgeCounter package (see Methods). (B,C) Binary heatmap comparing MACS2 called peaks under LD and DD conditions (B) and differentially accessible peaks at dawn, dusk, subjective dawn, and subjective dusk (C). (D) Distribution of subjective dusk and subjective dawn-accessible peaks with respect to known genomic features reported by ChIPpeakAnno. (E) Quantile-quantile plots showing correlation between ZT and CT peaks at dawn (ZT0/CT24) and dusk (ZT12/CT36). Differential peaks that are conserved between ZT and CT conditions (y-axis) shows lower adjusted p-values compared to peaks that are not conserved (i.e., only found in ZT, x-axis). (DOCX) [file pgen.1011278.s003.docx]

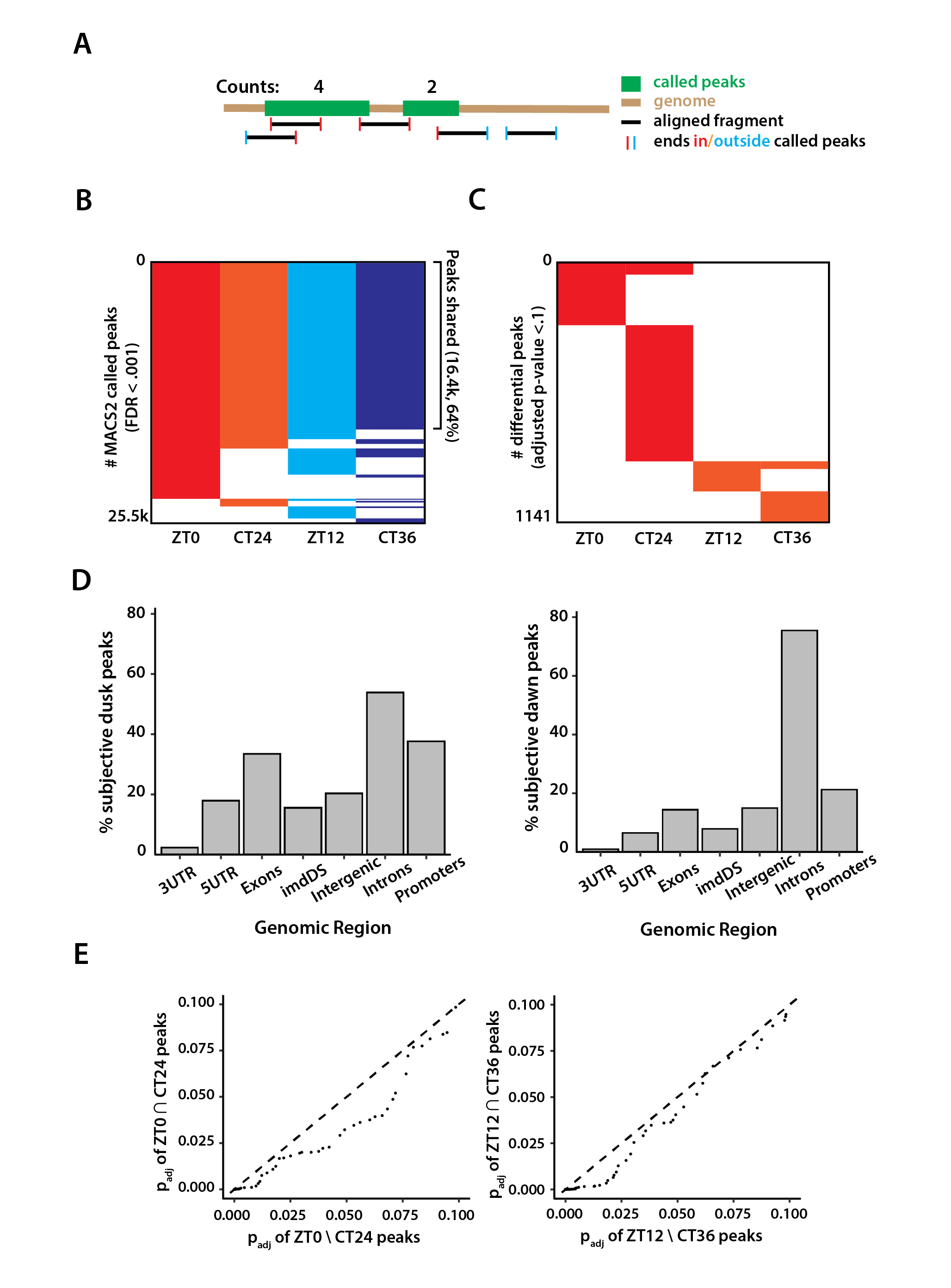


**S3 Fig. Schema of ATAC signal differential analysis.**

(**A**) Illustration of how we counted ATAC signal from MACS2 called peaks using the custom edgeCounter package (see Methods). (**B,C**) Binary heatmap comparing MACS2 called peaks under LD and DD conditions (B) and differentially accessible peaks at dawn, dusk, subjective dawn, and subjective dusk (C). (**D**) Distribution of subjective dusk and subjective dawn-accessible peaks with respect to known genomic features reported by ChIPpeakAnno. (**E**) Quantile-quantile plots showing correlation between ZT and CT peaks at dawn (ZT0/CT24) and dusk (ZT12/CT36). Differential peaks that are conserved between ZT and CT conditions (y-axis) shows lower adjusted p-values compared to peaks that are not conserved (i.e., only found in ZT, x-axis).
