# Supplementary material for: Clock-dependent chromatin accessibility rhythms regulate circadian transcription
Source: PLoS Genet. 2024 May 28;20(5):e1011278. doi: 10.1371/journal.pgen.1011278 (PMC11161047; doi:10.1371/journal.pgen.1011278)
Supplement: S4 Fig — (A,B) Clk locus is more accessible at dawn (ZT0/CT24) compared to dusk while per locus shows the opposite pattern. (C) Transcript variants of Pdp1 exhibit varying accessibility changes. The "long variants" possess a regulatory element that is more accessible at dusk, whereas the "short variants" display the opposite pattern. (D) vri locus also shows complex differential peak patterns. It possesses regulatory elements that are more accessible at dawn and dusk in GFP-positive clock neurons. Additionally, vri locus also possesses a regulatory element that is more accessible specifically at dusk even in non-clock cells (GFP-negative). Fold change and adjusted p-value are shown at the bottom of each of the differential peaks identified. (DOCX) [file pgen.1011278.s004.docx]

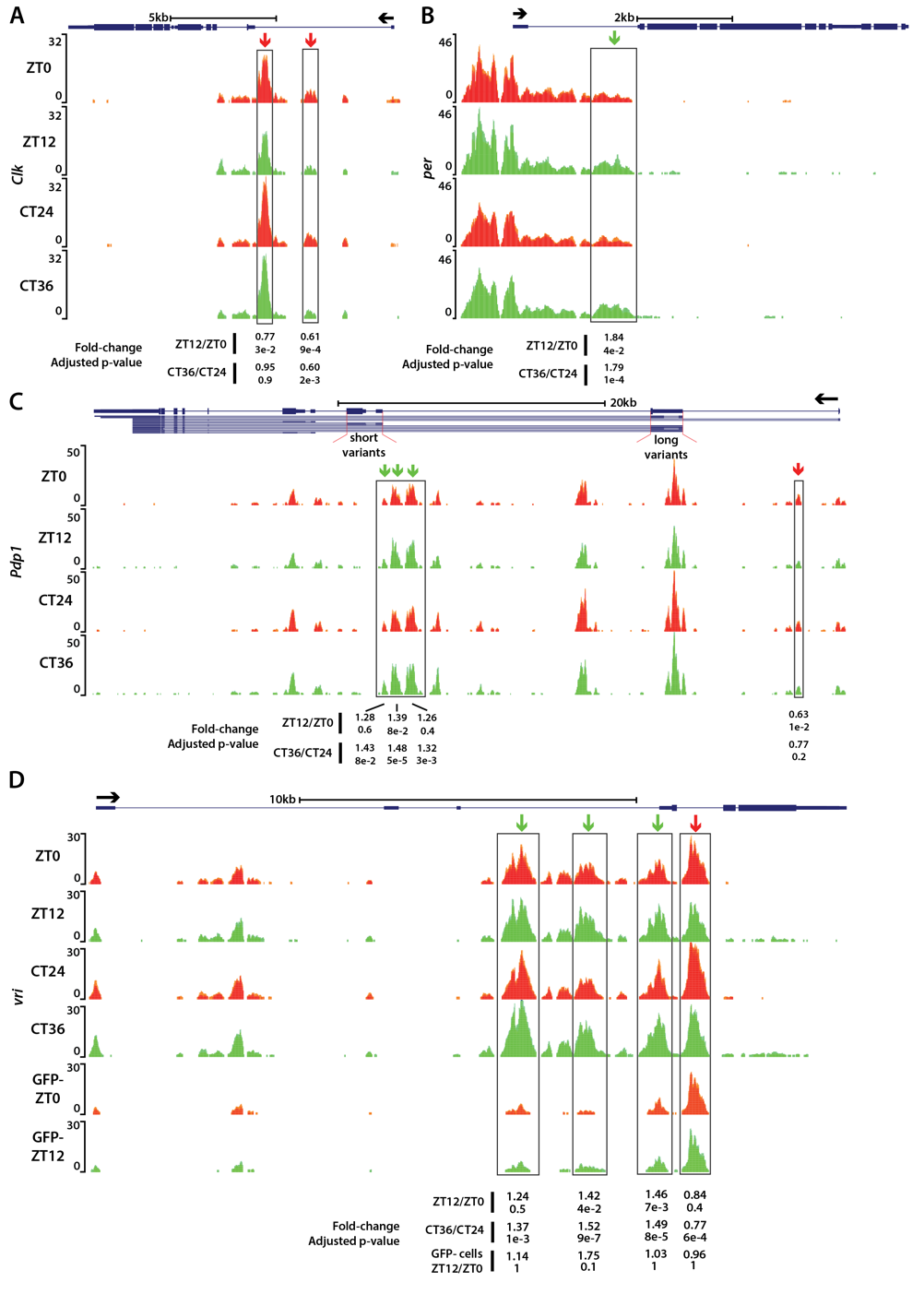


**S4 Fig. ATAC signal pile-up tracks of core clock genes *Clk, Pdp1 and vri* under LD and DD conditions.**

(**A,B**) *Clk* locus is more accessible at dawn (ZT0/CT24) compared to dusk while *per* locus shows the opposite pattern. (**C**) Transcript variants of Pdp1 exhibit varying accessibility changes. The "long variants" possess a regulatory element that is more accessible at dusk, whereas the "short variants" display the opposite pattern. (**D**) *vri* locus also shows complex differential peak patterns. It possesses regulatory elements that are more accessible at dawn and dusk in GFP-positive clock neurons. Additionally, *vri* locus also possesses a regulatory element that is more accessible specifically at dusk even in non-clock cells (GFP-negative). Fold change and adjusted p-value are shown at the bottom of each of the differential peaks identified.
