# Supplementary material for: Clock-dependent chromatin accessibility rhythms regulate circadian transcription
Source: PLoS Genet. 2024 May 28;20(5):e1011278. doi: 10.1371/journal.pgen.1011278 (PMC11161047; doi:10.1371/journal.pgen.1011278)
Supplement: S5 Fig — (A) List of genes that have differentially accessible peaks at both dusk and dawn. In all, 11 genes meet this criterion, and only those that exhibit cyclical changes in mRNA levels are presented here (6 out of 11). (B) Expression level of “short” and “long” transcript variants of Pdp1 in differential clock neuron subgroups (DN1, LNv, LNd) and a non-clock cell control (dopamine neurons, TH). While short variants show robust cycling profiles in all clock neuron groups, long variants are not cycling in DN1 neurons. Analysis was performed with publicly available data [28]. (DOCX) [file pgen.1011278.s005.docx]

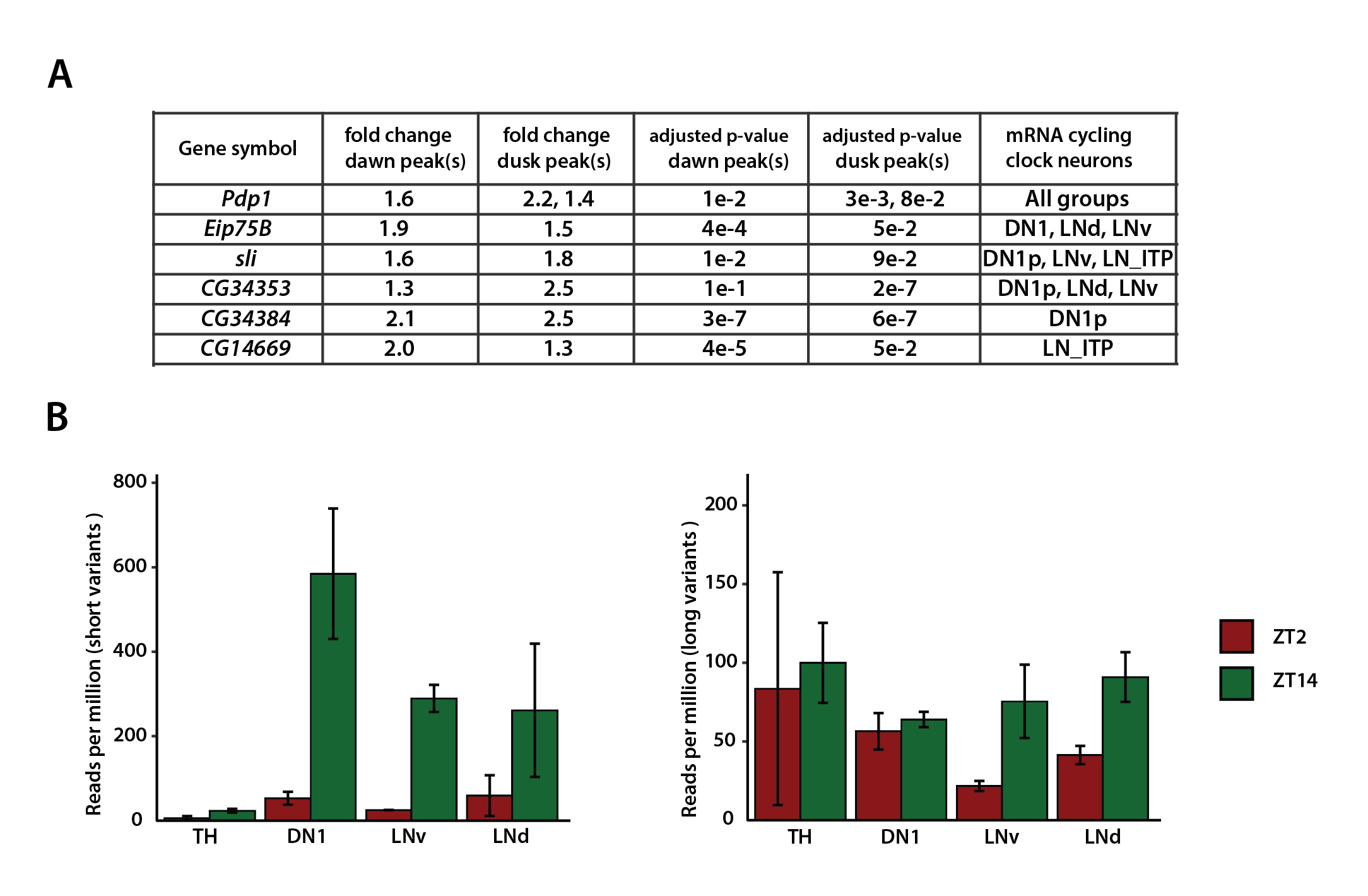


**S5 Fig. Genes with peaks that are more accessible at both dusk and dawn.**

(**A**) List of genes that have differentially accessible peaks at both dusk and dawn. In all, 11 genes meet this criterion, and only those that exhibit cyclical changes in mRNA levels are presented here (6 out of 11). (**B**) Expression level of “short” and “long” transcript variants of Pdp1 in differential clock neuron subgroups (DN1, LNv, LNd) and a non-clock cell control (dopamine neurons, TH). While short variants show robust cycling profiles in all clock neuron groups, long variants are not cycling in DN1 neurons. Analysis was performed with publicly available data (28).
