# Supplementary material for: Clock-dependent chromatin accessibility rhythms regulate circadian transcription
Source: PLoS Genet. 2024 May 28;20(5):e1011278. doi: 10.1371/journal.pgen.1011278 (PMC11161047; doi:10.1371/journal.pgen.1011278)
Supplement: S6 Fig — (DOCX) [file pgen.1011278.s006.docx]

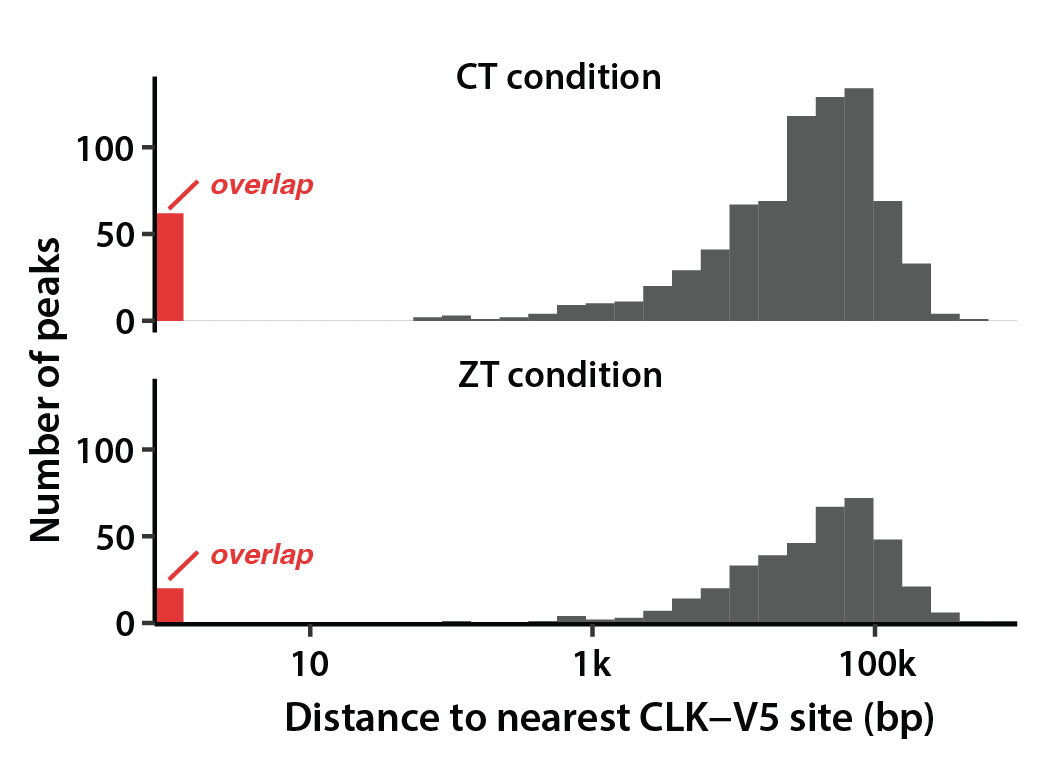


**S6 Fig. Overlap analysis of differential ATAC peaks to CLK-binding regions**

While some differential ATAC peaks overlap with CLK-binding regions identified in a previous microarray study (42), most (>90%) differential ATAC peaks are far away from CLK-binding regions with a median distance of ~30kb.
