# Supplementary material for: Clock-dependent chromatin accessibility rhythms regulate circadian transcription
Source: PLoS Genet. 2024 May 28;20(5):e1011278. doi: 10.1371/journal.pgen.1011278 (PMC11161047; doi:10.1371/journal.pgen.1011278)
Supplement: S7 Fig — (A) Representative images of Shaker intron 4 HCR-FISH in non-clock cells (fru-positive neurons). B) Quantification of fluorescence intensity of Shaker intron spot in fru-positive neurons. The statistical test used was a two-sided Student’s t-test, no significance was detected. Scale bar: 2μm. (DOCX) [file pgen.1011278.s007.docx]

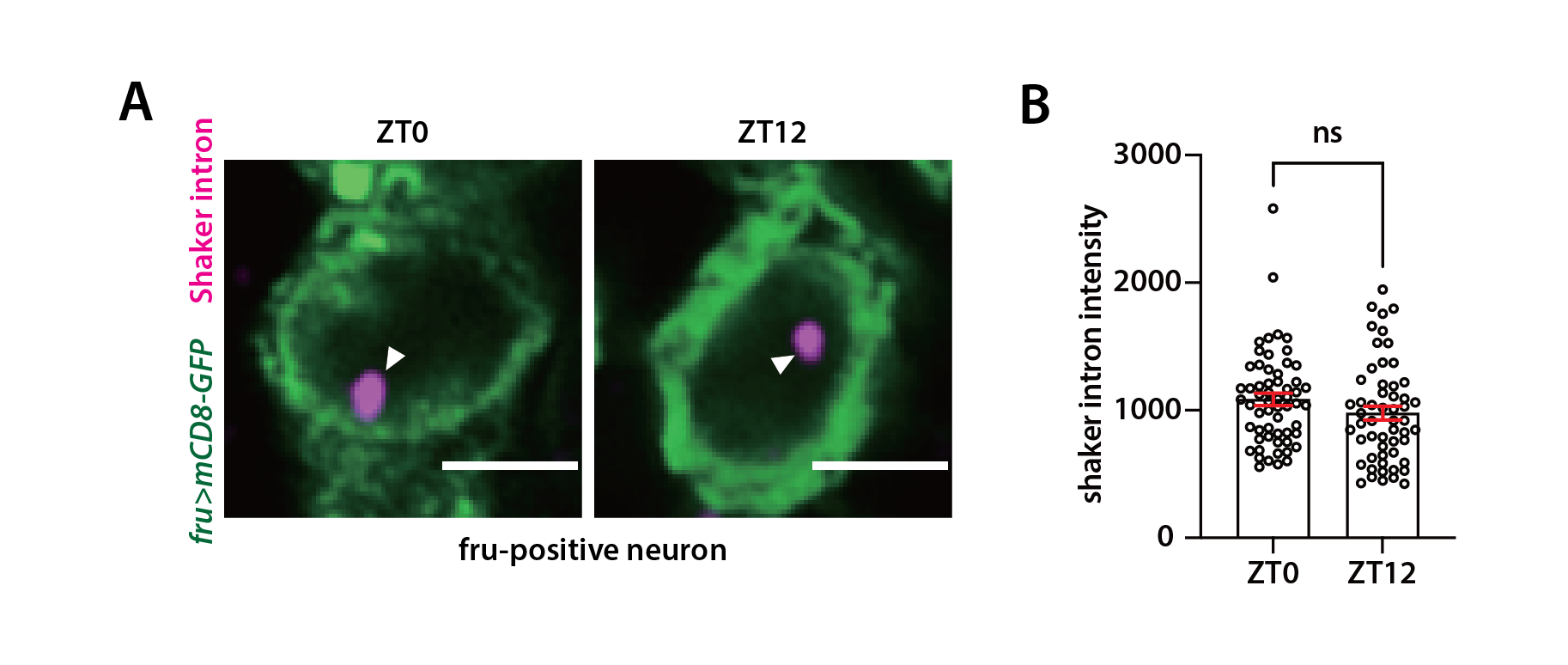


**S7 Fig. Transcriptional activity of *Shaker* in non-clock neurons.**

(**A**) Representative images of *Shaker* intron 4 HCR-FISH in non-clock cells (*fru*-positive neurons). B) Quantification of fluorescence intensity of Shaker intron spot in *fru*-positive neurons. The statistical test used was a two-sided Student’s t-test, no significance was detected. Scale bar: 2µm.
